# Analysis of plant expression profiles revealed that aphid attack triggered dynamic defense responses in sorghum plant

**DOI:** 10.3389/fgene.2023.1194273

**Published:** 2023-08-15

**Authors:** Yinghua Huang, Jian Huang

**Affiliations:** ^1^ USDA-ARS Plant Science Research Laboratory, Stillwater, OK, United States; ^2^ Department of Plant Biology, Ecology, and Evolution, Oklahoma State University, Stillwater, OK, United States; ^3^ Department of Plant and Soil Sciences, Oklahoma State University, Stillwater, OK, United States

**Keywords:** aphid, differential expression profiling, host plant defense, insect resistance, microarray, RNA-seq, sorghum, transcriptomics

## Abstract

Sorghum [*Sorghum bicolor* (L.) Moench] is one of the most important cereal crops grown worldwide but is often attacked by greenbug (aphid). In response to aphid attack, host plant initiates a large transcriptional reorganization, leading to activation of the host defense genes in aphid-attacked plants. In this study, our objective was to analyze defensive responses of sorghum against aphid and identify aphid resistance genes in sorghum. For the experiments, seedlings developed from an aphid resistant germplasm line (PI 550607) were divided into two groups, then, one group was infested with greenbug ((*Schizaphis graminum* Rondani) and the other group was used as control (un-infested). In addition, seedlings of sorghum cultivar Tx 7000, a susceptible genotype, prepared under the same conditions, were used as a genetic control. Those plant samples were used to develop transcriptional profiles using the microarray method, from which 26.1% of the 1,761 cDNA sequences spotted on the microarray showed altered expression between two treatments at 4 days after infestation. Sequence annotation and molecular analysis revealed that many differentially expressed genes (DEGs) were related to direct host defense or signal transduction pathways, which regulate host defense. In addition to common responsive genes, unique transcripts were identified in response to greenbug infestation specifically. Later, a similar transcriptional profiling was conducted using the RNA-seq method, resulted in the identification of 2,856 DEGs in the resistant line with a comparison between infested and non-infested at 4 days and 4,354 DEGs in the resistant genotype compared to the susceptible genotype at 4 days. Based on the comparative analysis, the data of RNA-seq provided a support for the results from the microarray study as it was noticed that many of the DEGs are common in both platforms. Analysis of the two differential expression profiles indicate that aphid triggered dynamic defense responses in sorghum plants and sorghum plant defense against aphid is a complex process involving both general defense systems and specific resistance mechanisms. Finally, the results of the study provide new insights into the mechanisms underlying host plant defense against aphids and will help us design better strategies for effectively controlling aphid pest.

## Introduction

Cereal aphids, phloem-feeding insects, are serious impediments to world food production [1, (Andrews et al., 1993; Porter et al., 1997)]. Some crops are damaged more than others, but all crops throughout the world are often attacked by at least one species of aphid. Greenbug (*Schizaphis graminum* Rondani) is a cereal crop aphid that has been recognized as a destructive pest of small grains, including major crops such as wheat, barley, sorghum, oat, corn, and even some wild and cultivated grasses (Burd, 2002; Royer et al., 2015). Heavy infestation of sap-sucking insects causes chronic shortage of photoassimilates, thus reducing the growth and production potential of the plants and ultimately resulting in plant death. In addition to the direct damages to plants, phloem-feeding insects are extremely effective vectors that facilitate the transmission of plant pathogens, viruses particularly, into the vascular tissue via stylet penetration during feeding.

Plant-aphid interaction is a complicated process. Indeed, greenbug aphids have an intimate and long-lasting interaction with plant tissues because the stylets of aphids that feed on phloem are in continuous contact with plant cells (Walling, 2000; Pontoppidan et al., 2003). It is not surprising to find that plant responses to phloem-feeding insects are different from responses to chewing insects. In fact, it has been proposed that the phloem-feeding insects are perceived somewhat like pathogens due to the similarities between the manner of penetration of plant tissues by fungal hyphae and aphid stylets (Pollard, 1973). While feeding, the aphids inject salivary secretion into plant tissues, which cause red or necrotic spots around the feeding areas (Pollard, 1973; Porter et al., 1997; Will and Vilcinskas, 2015). Little is known about the function of aphid salivary secretions. Phloem-feeding imposes important changes in phloem functions, which negatively affects growth and development of host plants (Girousse et al., 2003).

In the natural ecosystem, plants and aphids have co-evolved, meaning, for example, that standard plant barriers to aphid infestation can be circumvented by a particular aphid species or biotype, while otherwise successful aphids can also be blocked by the unique adaptive, defense mechanisms of certain resistant host plants (Mello and Silva-Filho, 2002). Host plants are also adapted to perceive the attack of insects as well as other stresses and to respond with self-protection. Aphids may trigger host plants to activate inducible defense responses, including activation of defense genes and signal pathways regulating inducible defense responses. Inducible defense mechanisms involve a broad range of proteins and other molecules whose synthesis is spatially and temporally controlled (Reymond et al., 2004; Liang et al., 2015) and which play roles in the recognition of attackers, signal transduction and protection against aphid attack. Phloem-feeding aphids are also able to induce the defense-signaling pathways commonly activated by bacterial, fungal, and viral pathogens (Fidantsef et al., 1999; Walling, 2000). Unfortunately, although aphid-plant interactions have been extensively studied, most research has concentrated on correlations between plant chemicals and aphid fitness, the basic mechanisms underlying such interactions and resultant defense responses remain largely unknown due to the complex nature of aphid feeding behavior. Thus, plant responses to phloem-feeding aphids are not as well understood as those to plant pathogens (Glazebrook, 2001) and even those to chewing insects (Reymond et al., 2000; Reymond et al., 2004).

With the recent advances in molecular biology and omics technologies, the transition to new biological research is apparent and genomic approaches are revolutionizing our understanding of plant-insect interaction. Gene expression profiling, including RNA-seq and microarray gene expression platforms, is a powerful tool to predict and interrogate mechanisms of host plant defense. Microarray emerged early and RNA-seq technology was developed recently and both platforms are useful for conducting transcriptional profiling (Rao et al., 2019). Furthermore, the tools and capacities of functional genomics (genome sequences, transcript profiling, and proteomics) are opening doors to new levels of studying plant responses to aphids. Studies of molecular defenses in response to attack by insects have been recently reported (Kessler and Baldwin, 2002; Garcia et al., 2021; Huang et al., 2022). Microarray, a powerful tool for gene discovery and expression profiling, has allowed significant progress in the study of plant-aphid interactions. A few reports described the molecular responses to greenbug attack in wheat (Zhang et al., 2020), sorghum (Grover, et al., 2022; Zhang et al., 2022) and switchgrass (Zogli et al., 2020). Sorghum-greenbug aphid interactions have received more attention lately and serve as a good system for examining defense of crop plants to cereal aphids. Zhu-Salzman et al. (2004) recently reported their comparative studies of transcriptional responses in a susceptible sorghum cultivar elicited either by greenbug, salicylic acid, or jasmonic acid. Our very recent report showed more comprehensive transcriptional profiles of a resistant sorghum cultivar in response to greenbug infestation (Park et al., 2006). These two studies well depicted the greenbug-responsive transcriptional profiles of commercial cultivars, but greenbug resistant mechanisms in wild type sorghum germplasm need to be explored. It is very intriguing that wild relatives of cultivated species have a diversity of defense strategies against a variety of stresses, including aphid pests. Thus, wild genotypes of sorghum may implement more diverse and certain novel resistance mechanisms for surviving attack by such enemies as greenbug aphids.

In this study, our primary goal was to identify defense genes activated by greenbug in a resistant wild genotype of sorghum (PI550607) and to assess unique resistance mechanisms operating in this genotype. Thus, the genome-wide expression profiles induced by greenbug were first developed using microarray and verified by RNA-seq. leading to identification of greenbug-responsive genes regulated by or associated with the incompatible interactions between resistant host plants and greenbug aphids. Now we present the data showing that both common defense and unique resistance mechanisms were activated in the plants in response to greenbug feeding.

## Materials and methods

### Preparation of plant seedlings and greenbug infestation

**FIGURE 1:**
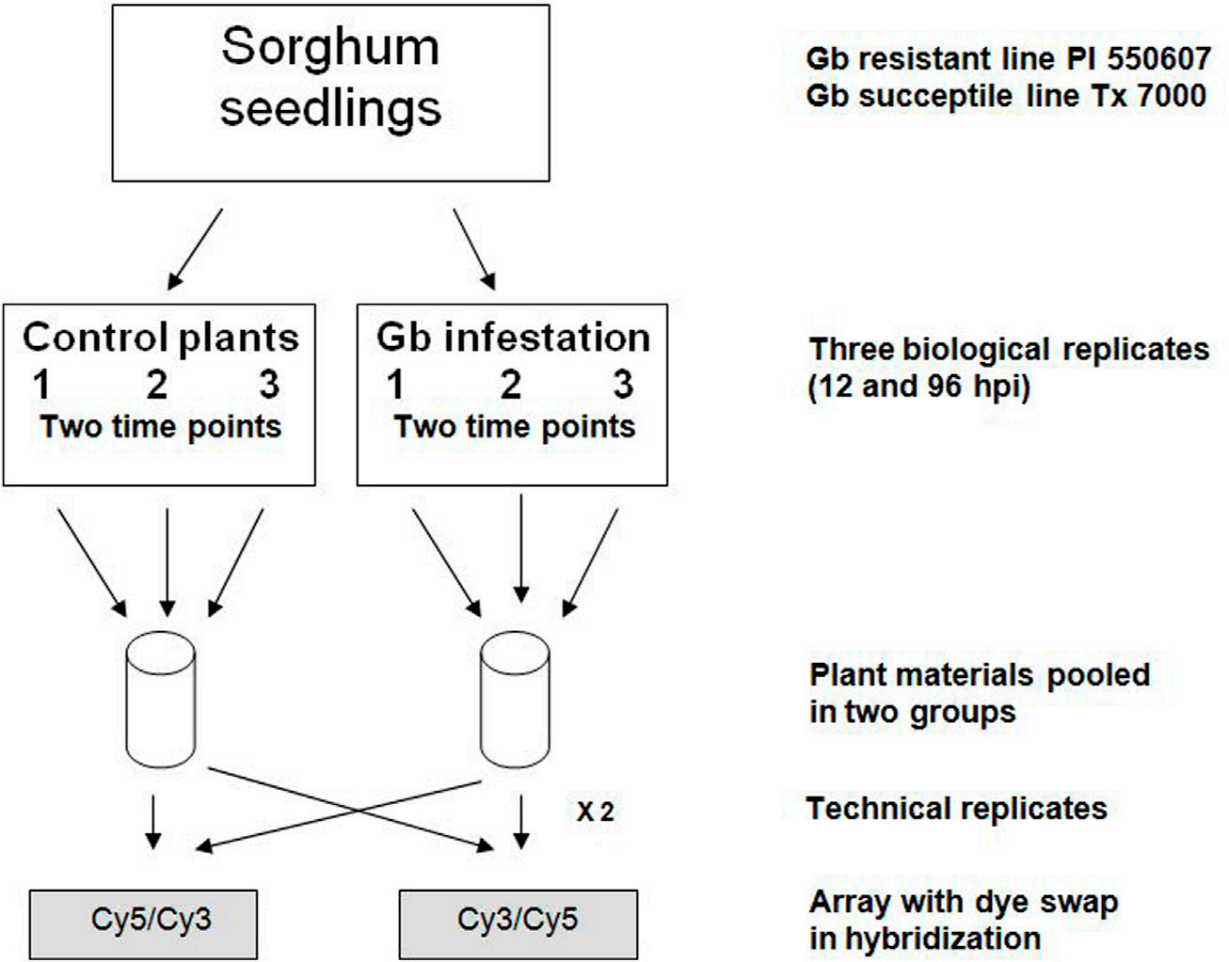
Experimental design and replicates for microarray analysis. Each sample of greenbug (Gb) infestation and control included three biological replicates and two technical replicates. Seedling tissues were collected at two different time points, 12- and 96-hour post-infestation (hpi).

A greenbug resistant genotype PI 550607 of sorghum [*Sorghum bicolor* (L.) Moench] identified from the sorghum germplasm collection (Andrews et al., 1993) and greenbug aphid [*S. graminum* (Rondani)] biotype I, a widespread virulent biotype on sorghum (Harvey et al., 1991) were used in this study. Sorghum seedlings and aphid cultures were prepared as previously reported (Park et al., 2006). For infestation treatment, seedlings at the 2-3 leaf stage were infested with 20 apterous adult aphids to the adaxial surface of the first true leaf. Then all seedlings and aphids were co-cultivated in 6-inch pots with clear plastic cage in the greenhouse at constant temperature (28°C ± 2°C) and 60% relative humidity under constant photoperiod of 14 h-light/10 h-dark (Park et al., 2006). Three biological and two technical replicates were included in each experiment, as described in Figure 1 to ensure that the resultant microarray data were sufficient to support and verify our conclusions. Infested seedlings were monitored and recorded for phenotypic changes or damage at the scheduled time points (Supplementary Figure S1). Then, seedling tissues above the soil were collected at two different time points (12- and 96-hour post infestation, hpi), quickly frozen in liquid nitrogen, and stored at −80°C until use. Non-infested seedlings were collected in the exact same way as the control. For phenotypic verification, two additional pots of infested and non-infested seedlings (20–30 each) were kept for symptom observation until 10 days post-infestation. In total, approximately 120–150 seedlings of a combination of three time-point collections were used for RNA extraction and microarray analysis. In addition, seedlings of sorghum cultivar Tx 7000, a susceptible genotype, prepared under the same conditions, were used as a control.

### RNA isolation and construction of cDNA library

RNAs were isolated from two pooled sorghum tissues, respectively, which were greenbug-infested and non-infested sorghum seedlings and the RNA was converted to cDNA as previously described (Park et al., 2006). Briefly, cDNAs obtained from the step described above were digested with restriction enzyme RsaI to obtain blunt-ends that are necessary for adaptor ligation. Equal quantities of poly(A)^+^ RNA derived from the greenbug-infested resistant plants and the non-infested plants were used for subtractive hybridization. cDNA subtraction (SSH) was carried out in two directions: forward subtracted cDNA library made of the pooled RNA of resistant plants as tester and the pooled RNA of susceptible plants as driver, and *vice versa* for the reverse subtracted library. The efficiency of cDNA subtraction was evaluated by comparing the expression levels of house-keeping genes, glyceraldehyde-3-phosphate dehydrogenase (GAPDH, XM_002439118, 5′-AAG​GCC​GGC​ATT​GCT​TTG​AAT, 3′-ACA​TGT​GGC​AGA​TCA​GGT​CGA) and the α-*Tubulin* gene (Sobic.001G107200, 5′-GAG​GTG​ACG​ATG​CTT​TCA​ACA​C, 3′-CAC​AGG​TCA​ACA​ATC​TCC​TTG​C), with other SSH subtracted cDNA fragments. The subtracted cDNA fragments were then inserted into the vector with the T/A cloning kit (Invitrogen, Carlsbad, California) and transformed into *E. coli* strain DH5α as the cDNA libraries.

### Microarray construction and hybridization

For construction of microarrays, a total of 1,761 cDNA sequences were amplified from plasmid clones derived from the cDNA library, As the previous report (Park et al., 2006), the resulted cDNA clones were purified, adjusted to a final concentration of 200 ng/ul, and then spotted onto amino-silane-coated slides (GAPSII coated slides, Corning Inc., Corning, New York) in triplicate by robotic spotting (PixSys 550 Microarrayer). The sorghum housekeeping genes (α-*Tubulin* and GAPDH) and three randomly selected, pre-tested sorghum cDNA clones were also printed on the same array to serve as internal controls. Negative controls on the array included 4 heterologous Arabidopsis genes, vector (PCR2.1) DNA without inserts, nested oligo primers, spotting buffer (3x SSC), and sterile water as blank. Each experiment was repeated twice with triplicate spots on each slide, resulting in six data sets.

For preparation of probes, each pool of mRNA from the two parallel samples (greenbug-challenged tissues and untreated control) was labeled with one of the two dyes using dendrimer-based methodology (the 3DNA Array 350 expression array detection kit, Genisphere, Pennsylvania). For microarray hybridization, 1 μg of mRNA was used to make cDNA probes for hybridization to the microarray. Two probes were made separately by reverse transcription of mRNA in the presence of either Cy5- or Cy3-labeled dUTP (Amersham, New Jersey) using SuperScript II (Invitrogen, Carlsbad, California). Microarrays were hybridized with the mixed Cy3 and Cy5 fluorescent-labeled probe pairs in a hybridization chamber (Corning, New York) as previously reported (Park et al., 2006).

### Acquisition and analysis of microarray data

The microarray slides were scanned in both 635 nm (Cy5) and 532 nm (Cy3) channels using a ScanArray Express Scanner (Perkin Elmer Applied Biosystems, Foster City, CA), and spot detection and quantification were carried out using GenePix Pro 4.0 software. Normalization of the data to equalize differences in Cy3-labeled and Cy5-labeled probe intensities was carried out using a correction factor obtained from the internal controls spotted on the array. Only useable spot pairs that passed normalization across all the biological replicates for each experiment were considered for further analysis. Replicate consistency checking was performed to remove hybridization spots giving poorly reproducible signals. Subsequently, the statistical values of relative abundance of individual transcripts were initially determined by the M statistic method. Transcripts showing relative expression changes in M value greater than 2.0 (mean log_2_-transformation ratios ≥ 1.0) or less than −2.0 (mean log_2_ ≤ −1.0) were considered either upregulated or downregulated. Furthermore, differential expression of genes was confirmed using an empirical Bayes method (the B-statistic) (Efron et al., 2001), and the mean values of these different replicates are given with standard errors (±SE). Genes that were not statistically significant (*p*-value < 0.05) were not considered to be differentially expressed.

### DNA sequencing and annotation

Nucleotide sequences of the differentially expressed cDNAs were determined using the ABI BigDye^TM^ termination cycle sequencing ready reaction kit and analyzed on an ABI Model 3700 DNA Analyzer (Perkin Elmer Applied Biosystems, Foster City, CA). All DNA sequences were compared with the Genbank databases through BLAST Network Service (National Center for Biotechnology Information, United States). Those Genbank homologs with highest scores were chosen to represent our cDNA clones and categorized by their biological function.

### RNA-seq analysis

For RNA-seq experiments, RNA from all the samples was prepared using the above-mentioned protocol and sent to Novogene Corporation Inc. (Sacramento, CA, https://en.novogene.com) for library construction and sequencing. These cleaned, high-quality reads were mapped to the latest version of the *S. bicolor* genome v3.1.1 available from Phytozome (https://phytozome-next.jgi.doe.gov/info/Sbicolor_v3_1_1) using HISAT2 software (Kim et al., 2015). The mapped reads were assembled using StringTie, and the subread program *featureCounts* (Liao et al., 2014) was used to count the read numbers mapped to each gene. The differential expression analysis was performed using the EdgeR package (Robinson et al., 2010) by comparing the infested samples to control for each genotype and time points. Those genes that showed log_2_ (fold change) ≥ 2 or ≤ −2 with a false discovery rate (FDR) adjusted *p*-value < 0.05 were considered as differentially expressed genes (DEGs). The sequencing was performed in Illumina Novoaseq platform (NovaSeq 6000) using a 150 bp paired-end strategy. Expression data (NVUS2021042933) were analyzed to explore the expression pattern and to determine the level of differentially expressed genes in response to aphid infestation. The other genes that showed low levels were removed and only the DEGs were used for further analysis.

### Northern blot analysis

For Northern blot analysis, total RNA samples were isolated as described above. RNAs were separated in gels by electrophoresis, blotted onto positively-charged nylon membrane (Hybond, Amersham, United States), and hybridized with gene-specific probes according to standard protocols (Sambrook and Russell, 2000). Probes were selected from the cDNA clones identified by microarray. Autoradiography was carried out on Kodak X-ray film.

### Quantitative real-time PCR (qRT-PCR)

For quantitative RT-PCR tests, an aliquot of the total RNA used for microarray analysis or RNA-seq was used for the reverse-transcription of each sample. Then, the resulted cDNAs were used for quantitative real-time PCR reactions which were performed with gene-specific primers and SYBR-Green reagents (Qiagen). The PCR program consisted of an initial polymerase activation step at 95°C for 3 min, 40 cycles of 30 s at 94°C, 30 s at 60°C, and 35 s at 72°C. Melt-curve analysis was performed to confirm the amplification of gene-specific products and monitor any non-specific amplification (Chou and Huang, 2010). Quantification was carried out using standard dilution curves for each gene and data were normalized based on the endogenous sorghum tubulin transcript. The average threshold cycle (*C*_*T*_) values calculated from triplicate reactions of each sample were used to determine the expression level relative to the control.

## Results

### Microarray analysis of sorghum challenged by greenbug

Greenbug, as a serious pest species on sorghum, has established compatible interactions with the host, although limited resistant sources of sorghum exist. To investigate molecular responses of resistant sorghum plants to greenbug feeding, the experiments were designed (Figure 1) to examine gene activities in young seedlings of a resistant sorghum genotype when challenged by biotype I greenbugs in comparison with control seedlings (i.e., the same genotype but un-infested). Greenbugs attempted to feed when they were placed on resistant seedlings. Regardless of the fate of greenbugs, attempting to feed affects expression of genes in the plants as the result of defense responses.

To capture a wide spectrum of differentially expressed genes, seedling tissues were harvested at 12 and 96 hpi and pooled before RNA extraction. RNA was also extracted from the non-infested seedlings of the same age, which were used as the driver (control) to facilitate construction of suppression subtractive hybridization (SSH) cDNA libraries. The efficiency test of the cDNA libraries using two sorghum housekeeping genes (*α-Tubulin*) and (GAPDH) indicated that these greenbug-regulated cDNAs were substantially enriched in the population of cDNA fragments used for SSH cDNA library construction, whereas transcripts of the housekeeping genes were dramatically reduced. In addition, the frequency of identical cDNA clones within libraries was low (approximately 10–15%). However, because the restricted cDNA fragments were used in the SSH procedure, two or more different cDNA fragments could represent a single transcript as reported elsewhere (Yang et al., 1999).

**FIGURE 2:**
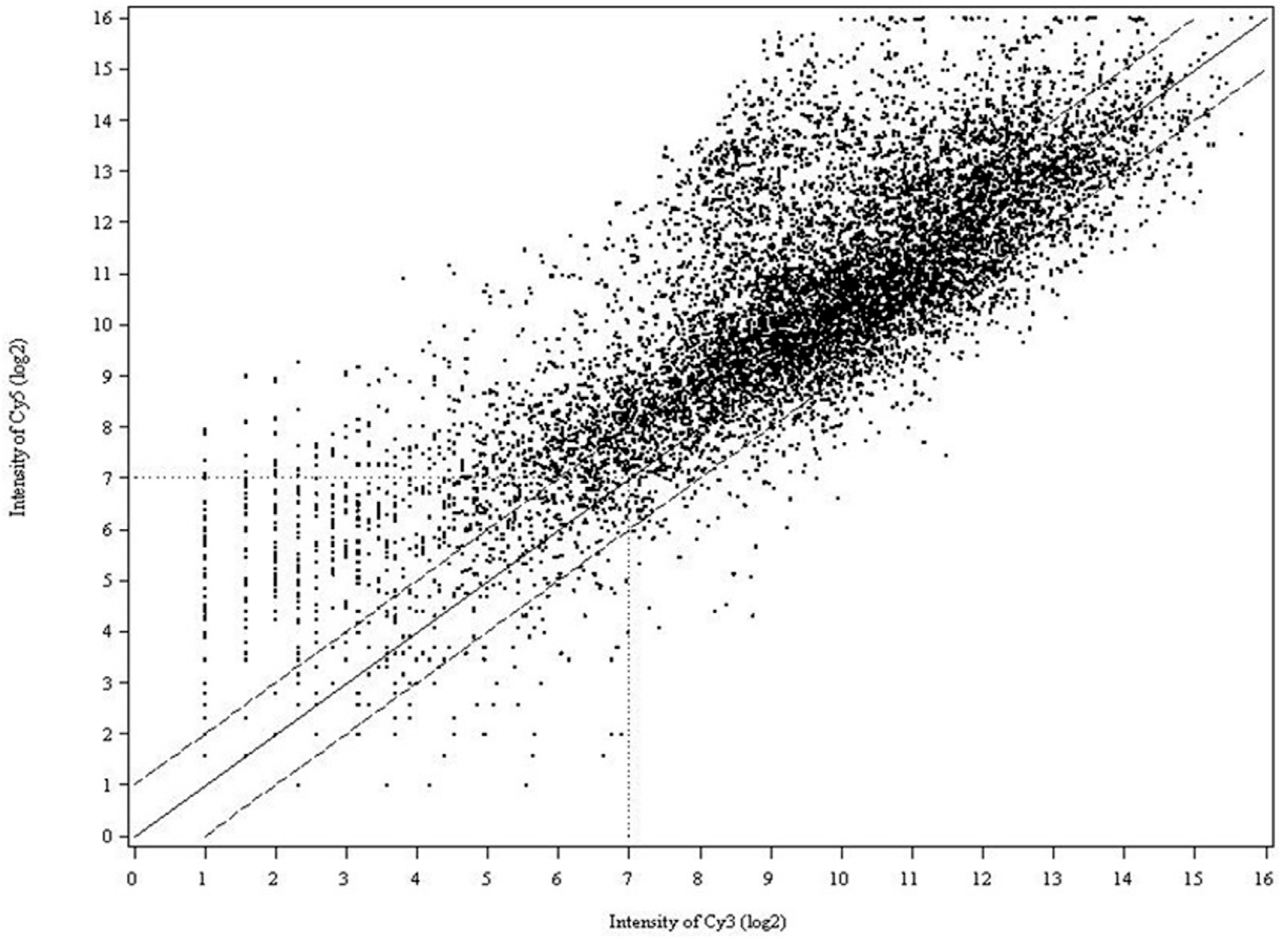
Scatter plot of spot intensity from the cDNA array on log2 scale where the signal from the Cy5 channel (tester) was plotted against the Cy3 channel (driver). Data from the images of both Cy3 and Cy5 were plotted as the mean signal intensity after normalization of the transcripts spotted in six replicates. The data points cluster around a ratio of 0, representing equal level of expression. The solid line (middle) marks a 1-fold increase (+) or decrease (−) in the Cy5/Cy3 ratio. The dashed lines indicate the 2-fold induction (or repression) cutoff level.

Three biological and two technical replicates were included in each experiment, as described in Figure 1 to ensure that the resultant microarray data were sufficient to support and verify our conclusions. In all experiments, we compared the intensities of Cy3- and Cy5-labeled probes to normalize variability. In competitive hybridizations with two samples, one labeled with Cy3, the other with Cy5 or *vice versa*, no notable difference in the resultant ratios could be observed (data are not shown). As shown in the scatter plot (Figure 2), differential expression patterns in the greenbug-infested sorghum were observed when the hybridization of cDNAs on the array to the probes from the tester (greenbug-challenged resistant genotype) was compared with the probes from the driver (non-infested same genotype). Results indicate that 459 genes were identified as aphid induced. Figure 2 also shows that a large number of genes (60.4%) were induced to express at higher levels; whereas a small number of genes (39.6%) were suppressed by greenbug feeding.

### Comparison RNA-seq and microarray expression profiles

**FIGURE 3:**
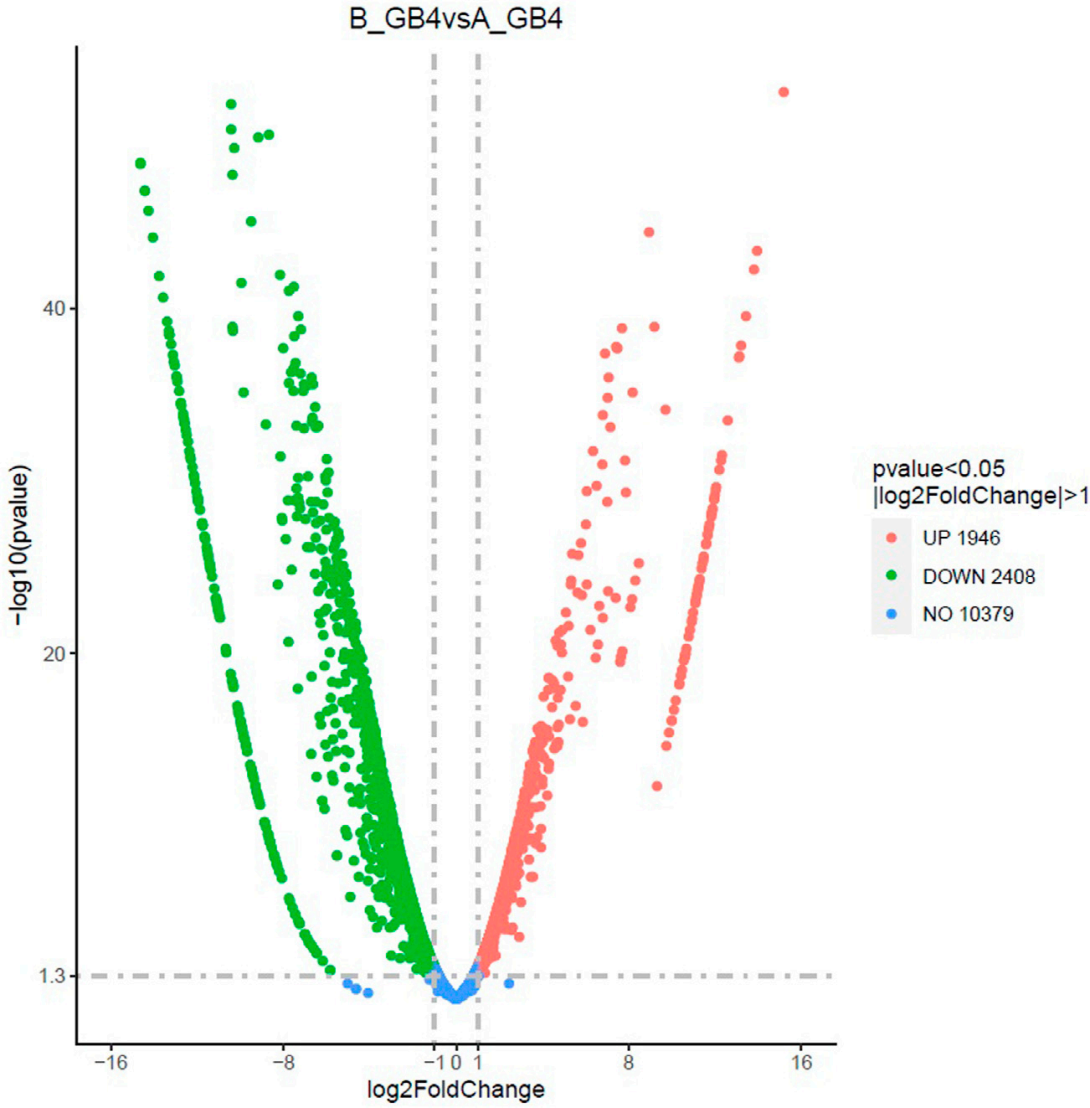
The volcano plot shows differentially expressed genes (DEGs) between the sorghum seedlings infested by greenbug and the control (un-infested seedlings) at the same stage. The *x*-axis shows the fold change in gene expression between different samples, and the *y*-axis shows the statistical significance of the differences. The significantly up- and downregulated genes are highlighted in red and green, respectively. Genes did not express differently between treatment group and control group are in blue.

**FIGURE 4:**
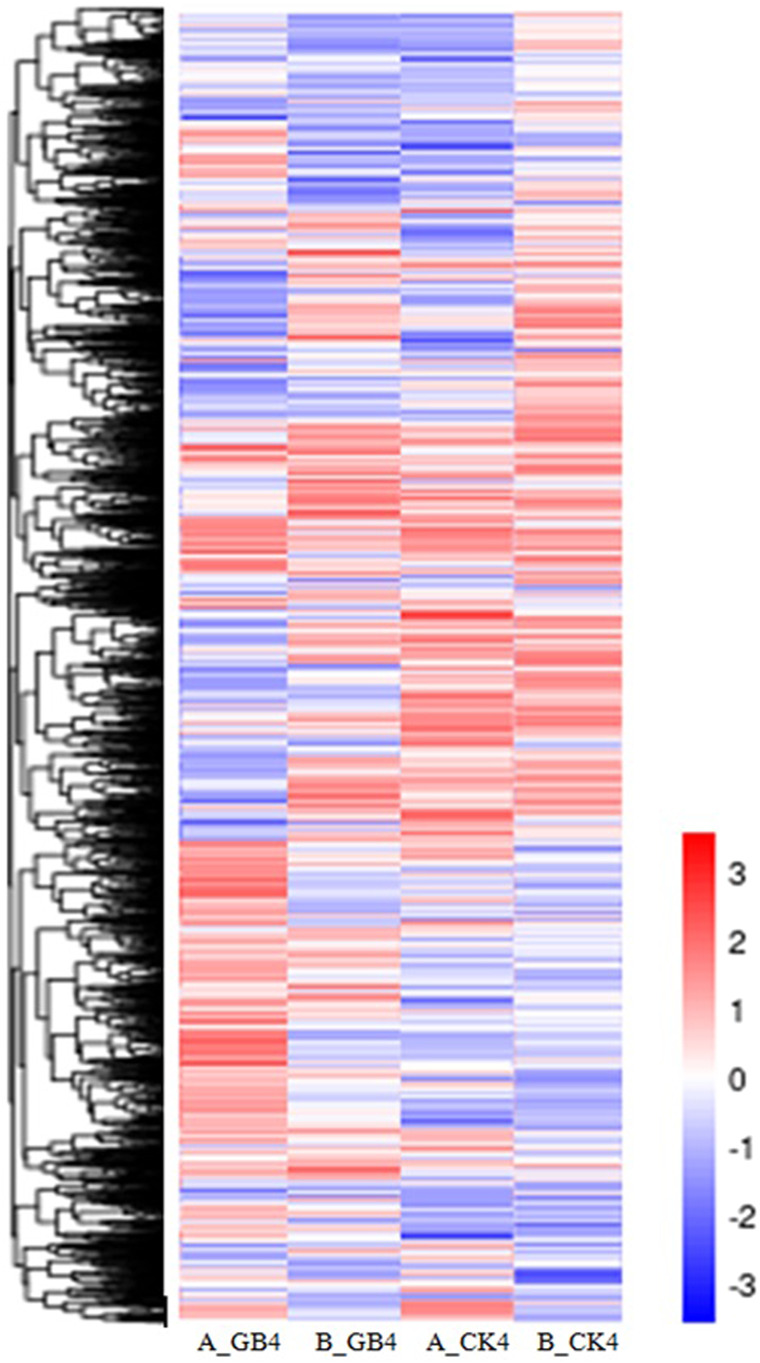
Heat map depicting changes in differential gene expression in response to aphid among various treatments, including the susceptible genotype infested by aphid at 4-dpi (A_GB4) and un-infested susceptible genotype (A_CK4), and the resistant genotype infested by aphid at 4-dpi (B_GB4) and un-infested resistant genotype (B_CK4). The values in the scale represent log2 fold change.

At the early stage, transcriptional profiles were developed using microarray platform, and then RNA-seq profiling was conducted with the same host plants under similar treatments. Analysis of the RNA-seq data revealed 4,352 (29.55% of total DEGs detected) genes in the resistant genotype compared to the susceptible one in response to aphid attack (Figure 3), which is a similar trend detected in the microarray results, but the patterns of differential expression depended on the genotypes that possess different ability to fight aphids. Heat map of the differential expression data shows the up- and downregulation of the aphid-responsive genes in the four treatments (Figure 4), which exhibited treatment-specific patterns. In the RNA-seq transcriptional profiles (Table 1), there were 1091 genes upregulated and 1765 downregulated (39.6%) in the resistant genotype PI 550607 at 4 days following aphid infestation when compared to those in non-infested plants. On the other hand, there were 2732 upregulated genes and 1791 downregulated genes in the susceptible genotype (Tx 7000) as shown in Table 1. Overall, the RNA-seq experiments provided quite similar results of aphid-induced DEGs from the microarray experiments though the setup and procedures of the two platforms were different. It was also noticed that the pattern of differential expression and dynamic range of expression for the differentially expressed genes with two platforms were in agreement between the expression profiling platforms. Thus, the RNA-seq results provided support for the microarray transcriptional profiling results. Comparison of the expression level of seven upregulated genes identified in the resistant genotype (PI 550607) shows the concordance of those genes between the RNA-seq and microarray platforms. These convincing results provided the evidence for newly identified genes that play the important role in sorghum for self-protection from aphid attack.

**TABLE 1:** List of umbers of differentially expressed genes (DEGs) in the sorghum seedlings among three different treatments at 4 days after infestation by the virulent greenbug biotype I.

	A_GB4 vs. A_CK4	B_GB4 vs. B_CK4	B_GB4 vs. A_GB4
Total number of transcripts identified	14,677	14,687	14,733
No significant change in expression	10,154	11,831	10,379
**Transcripts with altered expression level (DEGs)**	**4,523**	**2,856**	**4,354**
% of DEGs in total transcripts identified	30.83	19.45	29.55
Upregulated transcripts	2,732	1,091	1,946
% of upregulated in the total DEGs	60.4	38.2	44.69
Downregulated transcripts	1,791	1,765	2,408
% of downregulated in the total DEGs	39.6	61.8	55.31

Note: A_GB4 represents the susceptible genotype (Tx 7000) co-cultivated with greenbug for 4 days (96 hpi), A_CK4 for the non-infested Tx 7000 after 4 days, B_GB4 for the resistant genotype (PI 550607) co-cultivated with greenbug for 4 days (96 hpi), and B_CK4 for the non-infested resistant genotype (PI 550607) after 4 days.

That the bold values indicate the genes with differential expression at significant levels as stated in the column A of the table.

### Large-scale changes in gene activities in response to greenbug attack

In Table 2, microarray results showed comprehensive gene activation in sorghum seedlings that were challenged by greenbugs. Many of these genes are related to either direct defense or signal transduction pathways (i.e., for gene regulation) as well as functions involved in re-routing metabolism into the production of defensive compounds. The expression ratios of transcripts between two identical mRNA samples isolated from infested and non-infested tissues were measured. We examined the expression changes that occurred in the abundance of transcripts corresponding to all cDNA clones (SSH inserts) printed on the arrays. Among the 1,761 cDNA clones, genes showing either induced or suppressed expression levels in greenbug-challenged seedlings relative to non-infested seedlings were identified. If the intensity ratio (Cy5/Cy3) of a gene showed ≥2.0-fold changes (in either induction or suppression), the gene is considered to be differentially regulated by greenbug infestation. The significant level of transcript abundance was also confirmed using the B-statistical analysis (Efron et al., 2001). Using these filters, 459 cDNA clones were identified as differentially expressed gene products, representing 26.1% of 1,761 cDNA sequences printed on the arrays. The numbers of greenbug-regulated transcripts at various expression levels are shown in Supplementary Figure S2. Among these greenbug-regulated genes, there were 448 genes with expression increased and 11 genes reduced by two folds or more. Interestingly, many genes were induced to express at considerably higher levels, whereas only a small number of genes were suppressed by greenbug feeding in this resistant sorghum genotype (Table 2).

**TABLE 2:** Aphid-responsive genes identified from sorghum plants (PI 550607, an aphid-resistant genotype) showing differential expression in response to artificial infestation with phloem-feeding greenbug.

Clone id	Putative function/homology/species^a^	Fold changes^b^ identified by RNA-seq	Fold changes^b^ identified by microarray	Accession no.
1	Cell maintenance and cell wall fortification			
P1-G23	Proline-rich protein APG_*Oryza sativa*	1.6810845	3.201 ± 0.954	DV162782
P1-M23	Proline-rich protein APG_*Oryza sativa*	1.6810845	3.191 ± 1.056	DV162759
P2-H12	Cellulase_*Medicago truncatula*	-	−0.641 ± 0.203	DV162774
P2-J5	Proline-rich protein APG_*Oryza sativa*	1.6810845	3.393 ± 0.723	DV162817
P3-G17	Proline-rich protein APG_*Oryza sativa*	1.6810845	3.518 ± 0.267	DV162797
P4-C23	Proline-rich protein APG_*Oryza sativa*	1.6810845	3.761 ± 0.707	DV162798
P4-J10	ZmGR1b (Proline-rich protein)_*Zea mays*	4.071132928	−1.122 ± 0.282	DV162800
P4-L1	ZmGR1b (Proline-rich protein)_*Zea mays*	4.071132928	−1.020 ± 1.035	DV162801
P5-D1	Ribosomal Protein 40S S2_*Oryza sativa*	-	1.199 ± 0.359	DV162796
2	Defense-related proteins			
P1-B9	Sulfur-rich thionin-like protein_*Triticum aestivum*	4.031698866	3.947 ± 1.129	DV162839
P1-D16	Sulfur-rich thionin-like protein_*Triticum aestivum*	4.031698866	4.042 ± 0.985	DV162832
P1-L16	Sulfur-rich thionin-like protein_*Triticum aestivum*	4.031698866	3.881 ± 1.111	DV162831
P2-A18	B-1,3-glucanase_*Oryza sativa*	2.569910291	3.219 ± 1.111	DV162830
P2-I11	3′exoribonuclease_*Oryza sativa*	-	2.604 ± 0.228	DV162741
P2-L21	Sulfur-rich thionin-like protein_*Triticum aestivum*	4.031698866	3.632 ± 0.932	DV162836
P2-O6	Sulfur-rich thionin-like protein_*Triticum aestivum*	4.031698866	3.846 ± 1.140	DV162834
P3-A12	D-serine deaninase activator_*Escherichia coli*	-	−0.868 ± 0.361	DV162813
P3-D22	Sulfur-rich thionin-like protein_*Triticum aestivum*	4.031698866	3.078 ± 0.826	DV162833
P3-E22	Sulfur-rich thionin-like protein_*Triticum aestivum*	4.031698866	3.436 ± 0.886	DV162838
P3-J15	Pathogenesis-related protein-5_*Zea mays*	2.500528214	2.906 ± 0.389	DV162815
P3-J21	Bromelian-like thiol protease_*Oryza sativa*	4.345336973	3.847 ± 0.113	DV162744
P3-J22	Mannose Binding Lectin Precursor_*Rhodinus prolixus*	-	1.654 ± 0.285	DV162740
P3-N14	Glucan endo-1,3-B-glucosidase_*Zea mays*	-	2.405 ± 1.453	DV162843
P3-O3	Polyphenol oxidase PPO1_*Populus balsmifera*	-	3.199 ± 0.769	DV162850
P4-A19	Sulfur-rich thionin-like protein_*Triticum aestivum*	4.031698866	3.943 ± 1.107	DV162835
P4-A22	Sulfur-rich thionin-like protein_*Triticum aestivum*	4.031698866	3.590 ± 0.947	DV162841
P4-G8	Bromelian-like thiol protease_*Oryza sativa*	4.345336973	3.740 ± 0.822	DV162840
P4-F11	Cyanogenic B-glucosidase d hurrinase-2_*Sorghum bicolor*	-	2.240 ± 0.669	DV162745
P4-F7	Thaumatin-like protein_*Triticum aestivum*	2.500528214	3.148 ± 0.260	DV162808
P4-I16	Class III Chitinase_*Sphenostylis stenocarpa*	-	3.443 ± 0.759	DV162767
P4-J18	Sulfur-rich thionin-like protein_*Triticum aestivum*	4.031698866	3.795 ± 0.558	DV162842
P4-O22	Sulfur-rich thionin-like protein_*Triticum aestivum*	4.031698866	3.754 ± 0.175	DV162763
P4-O7	Latex allergen hev b 7.02_*Hevea brasiliensis*	-	2.993 ± 0.342	DV162794
P5-C8	Bromelian-like thiol protease_*Oryza sativa*	4.345336973	3.734 ± 0.482	DV162837
P5-D14	Bet v I allergen _*Zea mays*	-	−1.223 ± 0.900	DV162748
P5-H12	Putative dipeptidyl peptidase IV_*Oryza sativa*	-	2.611 ± 0.904	DV162792
P5-H16	Sulfur-rich thionin-like protein_*Triticum aestivum*	4.031698866	4.463 ± 0.568	DV162844
P5-J11	Sulfur-rich thionin-like protein_*Triticum aestivum*	4.031698866	3.685 ± 0.921	DV162742
P5-L17	Sulfur-rich thionin-like protein_*Triticum aestivum*	4.031698866	4.436 ± 0.501	DV162845
3	Growth and development			
P1-I11	Auxin-repressed protein_*Manihot esculenta*	-	3.371 ± 0.768	DV162781
P2-J6	Auxin-repressed protein_*Manihot esculenta*	-	1.269 ± 0.150	DV162805
4	Metabolism			
P1-B14	Aminoacid transporter_*Magnetospirillum magnetotacticum MS-1*	-	3.923 ± 0.640	DV162760
P1-F18	ABC transporter ATP binding protein_*Mycoplasma hyopneumoniae 232*	-	2.750 ± 0.808	DV162790
P1-J21	Methyltransferase_*Oryza sativa*	-	−0.585 ± 0.719	DV162776
P1-N6	ADP/ATP translocase_*Anopheles gambiae*	-	2.289 ± 0.337	DV162791
P2-B5	Alpha tubulin _*Oryza sativa*	-	−0.744 ± 0.340	DV162775
P2-F11	Golgin-84_*Homo sapiens*	2.437693551	3.139 ± 0.547	DV162821
P3-A17	Mitochondrial aldehyde dehydrogenase_*Arabidopsis thaliana*	-	1.024 ± 0.195	DV162802
P3-G6	Alcohol dehydrogenase _*Arachis hypogaea*	2.437693551	2.951 ± 0.752	DV162820
P3-I13	ADP/ATP translocase_*Anopheles gambiae*	-	1.169 ± 0.345	DV162825
P3-I18	Inorganic diphosphatase_*Hordeum vulgare*	-	−1.024 ± 0.106	DV162752
P4-K16	Arginine decarboxylase_*Malus x domestica*	-	2.040 ± 0.448	DV162788
P5-A14	Phosphoethanolamine N-methyltransferase_*Zea mays*	-	1.642 ± 0.257	DV162786
5	Photosynthesis and housekeeping			
P2-H16	Chloroplast hypothetical protein_*Zea mays*	-	−1.434 ± 0.418	DV162751
P3-A18	Ferredoxin_*Zea mays*	−1.046216821	0.239 ± 0.274	DV162846
P5-L9	Oligosaccharyl transferase STT3_*Oryza sativa*	-	1.166 ± 0.237	DV162780
6	Regulators			
P1-O15	Translation initation factor 2 alpha subunit eIF2_*Arabidopsis thaliana*	-	−1.192 ± 0.063	DV162770
P2-N1	LHY protein_*Oryza sativa*	−1.424339346	1.032 ± 0.264	DV162824
P3-B3	Homeobox protein Hox 11/13_*Heliocidaris erythrogramma*	-	−0.825 ± 0.493	DV162777
P4-B9	Zinc finger-like protein_*Oryza sativa*	3.744605696	2.005 ± 0.658	DV162787
P4-D9	Zinc finger-like protein_*Oryza sativa*	3.744605696	3.072 ± 0.687	DV162809
P4-P11	Zinc finger-like protein_*Oryza sativa*	3.744605696	2.864 ± 0.329	DV162827
7	Stress response			
P1-N10	Glutathione S-transferase II _*Oryza sativa*	-	2.786 ± 0.924	DV162814
P1-O22	Catalase_*Campylobacter jejuni*	-	3.624 ± 0.924	DV162816
P3-B23	DNAJ-like protein_*Orzya sativa*	-	1.011 ± 0.214	DV162784
P5-B11	Catalase_*Campylobacter jejuni*	-	3.130 ± 0.809	DV162819
8	Signal transduction			
P1-D24	Patatin-like protein_*Sorghum bicolor*	2.437693551	4.225 ± 0.706	DV162754
P1-E13	Family II Lipase EXL4_*Oryza sativa*	-	−1.004 ± 0.089	DV162769
P1-K14	GDSL-lipase_*Oryza sativa*	1.6810845	4.298 ± 0.276	DV162764
P1-L22	Patatin-like protein_*Sorghum bicolor*	2.437693551	4.001 ± 0.749	DV162757
P1-L6	Patatin-like protein_*Sorghum bicolor*	2.437693551	3.260 ± 0.776	DV162811
P1-L14	Patatin-like protein_*Sorghum bicolor*	2.437693551	3.560 ± 0.906	DV162766
P1-M15	Patatin-like protein 3_*Nicotiana tabacum*	2.437693551	3.301 ± 0.000	DV162795
P1-M19	Patatin-like protein_*Sorghum bicolor*	2.437693551	3.652 ± 0.725	DV162758
P2-C14	Patatin-like protein_*Sorghum bicolor*	2.437693551	4.549 ± 0.147	DV162755
P2-P4	Lipoxygenase_*Oryza sativa*	2.563951662	2.774 ± 0.683	DV162807
P2-K23	Patatin-like protein_*Sorghum bicolor*	2.437693551	3.597 ± 0.749	DV162768
P2-N2	Family II Lipase EXL4_*Oryza sativa*	−9.066797155	−1.941 ± 1.320	DV162747
P3-B11	Patatin-like protein_*Oryza sativa*	2.437693551	3.944 ± 0.755	DV162812
P3-C13	Patatin-like protein_*Oryza sativa*	2.437693551	2.923 ± 0.765	DV162829
P3-G21	Patatin-like protein_*Sorghum bicolor*	2.437693551	3.610 ± 0.687	DV162765
P3-H16	Patatin-like protein_*Sorghum bicolor*	2.437693551	2.932 ± 0.687	DV162793
P3-I7	Patatin-like protein_*Sorghum bicolor*	2.437693551	4.131 ± 0.533	DV162756
P3-N1	Lipoxygenase_*Zea mays*	-	2.150 ± 0.766	DV162743
P4-K7	Lipoxygenase 2_*Two-rowed barley*	-	2.255 ± 0.673	DV162789
P4-L14	Patatin-like protein_*Sorghum bicolor*	2.437693551	4.115 ± 0.422	DV162762
P4-M6	Patatin-like protein_*Sorghum bicolor*	2.437693551	4.536 ± 0.163	DV162753
P4-N22	Map kinase phosphatase-like MK-STYX_*Homo sapiens*	-	1.917 ± 0.396	DV162804
P5-C3	Lipoxygenase 2.3_*Oryza sativa*	-	1.368 ± 0.483	DV162778
P5-J1	Papain-like protein SPE31_*Pachyrhizus erosus*	2.437693551	3.832 ± 0.851	DV162761
P5-L8	Lipoxygenase 2_*Hordeum vulgare*	-	1.702 ± 0.505	DV162779
9	Others			
P1-H20	XptA2 protein_*Xenorhabdus nematophilia*	−1.487831178	2.006 ± 0.480	DV162746
P2-O21	tnp protein-transposon Tn4451_*Clostridium perfringes*	−2.200418507	−0.594 ± 0.850	DV162750
P4-C12	Kinesin motor related protein_*Oryza sativa*	−1.324286979	1.398 ± 0.304	DV162818
P4-I7	Complement component 3_*Mus musculus*	-	1.643 ± 0.290	DV162803
P5-A2	TatD Dnase domain containing 1_*Canis familiaris*	-	2.346 ± 0.654	DV162826
P5-C11	Capsid protein _*Tomato chlorotic mottle virus*	3.744605696	3.366 ± 1.015	DV162806
10	Unknown function			
P1-L23	Unknown protein _*Oryza sativa*	-	−0.815 ± 0.591	DV162749
P1-M9	Unknown protein _*Schistosoma japonicum*	-	3.012 ± 0.518	DV162828
P1-O18	Hypothetical protein_*Neurospora crassa*	-	−0.877 ± 0.512	DV162848
P3-G13	Unknown Protein _*Arabidopsis thaliana*	-	1.619 ± 0.236	DV162822
P3-H22	Unnamed protein product_*Kluyveromyces lactis*	2.437693551	3.765 ± 0.753	DV162799
P4-H4	Hypothetical protein_*Leishmania major*	-	−1.188 ± 0.079	DV162783
P5-J8	Hypothetical protein_*Rickettsia typhi str. Wilmington*	-	1.162 ± 0.188	DV162849
P1-L12	No hit	-	3.456 ± 0.588	DV162785
P1-P21	No hit	-	1.914 ± 0.179	DV162823
P2-K10	No hit	-	−1.162 ± 1.723	DV162847
P2-L13	No hit	-	−0.870 ± 1.723	DV162771
P3-D5	No hit	-	−0.037 ± 1.723	DV162772
P4-B5	No hit	1.6810845	3.081 ± 0.604	DV162810
P5-L6	No hit	-	−0.855 ± 0.359	DV162773

^a^ Gene functional annotation with maximum homology was determined by a BLAST search of the public Genbank databases. The scores for the closest protein of known function as identified from the BlastX or BlastN search are shown in the next two columns.

^b^ Ratios of signal intensity were based on the cDNA microarray hybridization data, comparing expression levels between greenbug-infested and non-infested tissues. Mean expression ratios (±SD) were calculated from nine replicates for comparison of greenbug-challenged and unchallenged plants.

**FIGURE 5:**
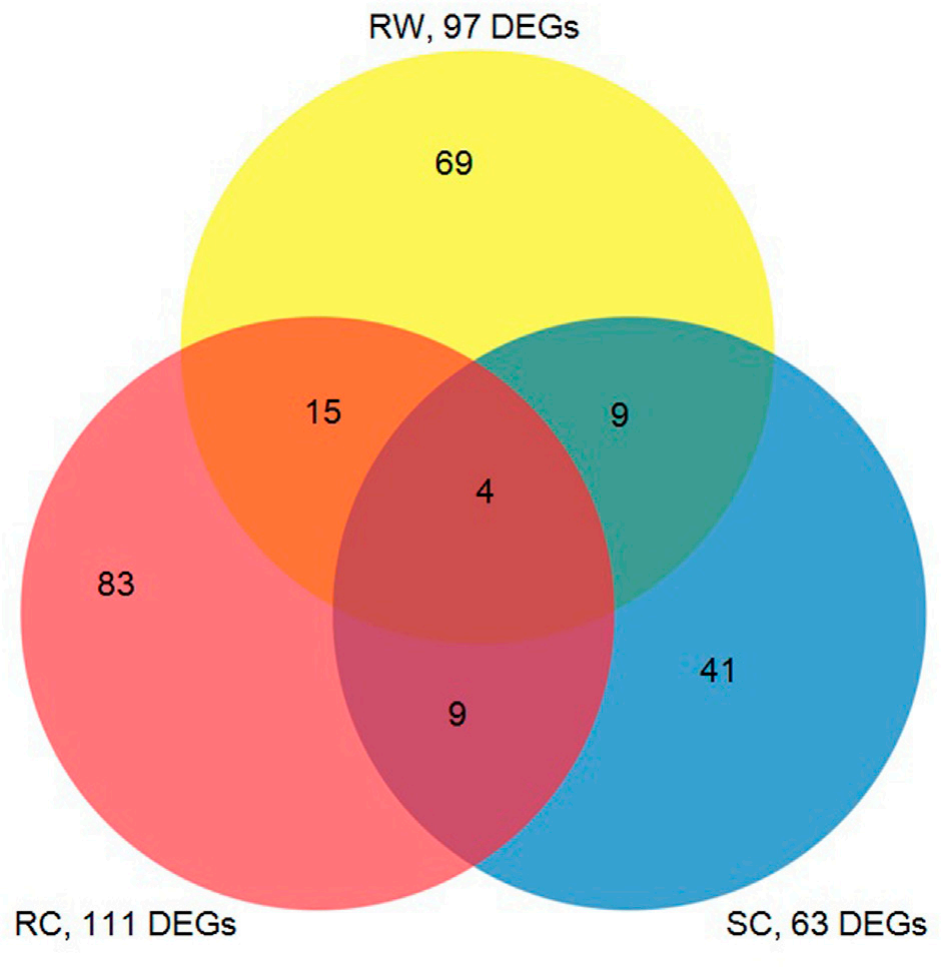
The venn diagram represents the number of transcripts in response to infestation by virulent greenbugs showing common or differential expression among a susceptible sorghum cultivar (SC, Tx 7000), a resistant cultivar (RC, M627), and a resistant wild type sorghum germplasm line (RW, PI550607) used in this study. The numbers of cDNAs in the overlapping areas indicate the co-expressed transcripts in comparisons of three unique lines.

The DEG data generated from this study were compared with the previous results of the microarray experiments (Park et al., 2006), showing both common responses and unique responses of three sorghum genotypes, which including the susceptible cultivar (Tx 7000) and the wild gerplasm (PI 550607) in this study as well as a resistant commercial hybrid (M627) from our previous study (Park et al., 2006). The Venn diagram (Figure 5) reveals the number of highly expressed defensive genes among the three genotypes in response to the same clone of greenbug biotype I. It is believed that the aphid-induced transcripts shared by the three lines belong to the genes associated with the general defense response; otherwise they may relate to the unique resistance factor(s) in the specific line.

**FIGURE 6:**
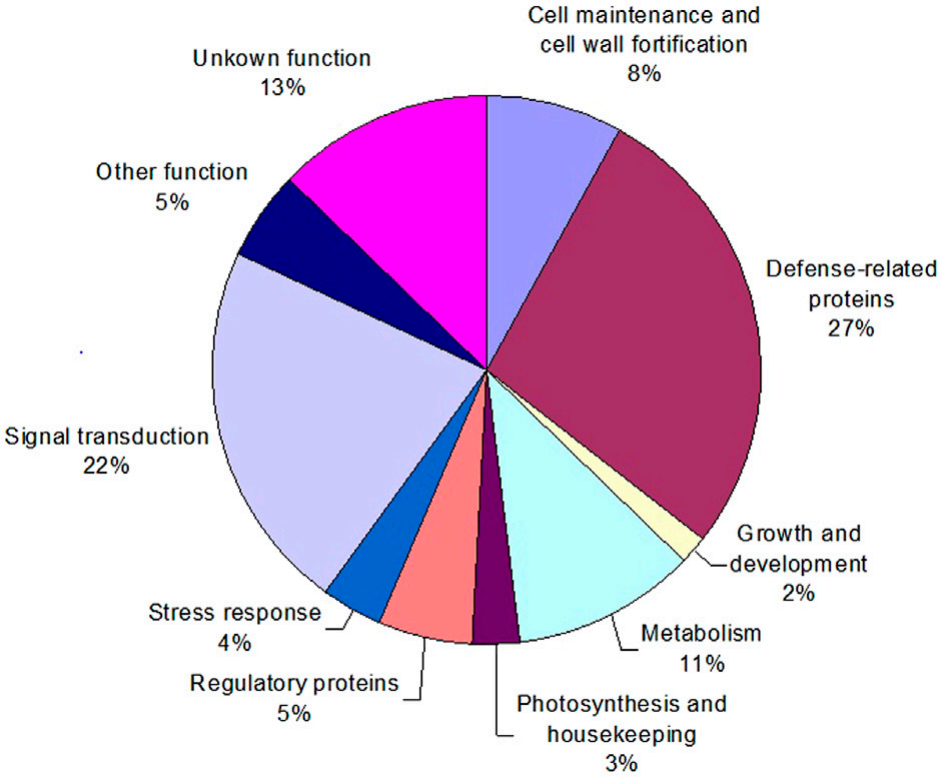
Distribution of functional categories of the identified sorghum genes responsive to greenbug challenge. The percent values represent each functional category of the total responsive genes (DEGs).

To characterize sorghum genes whose products are involved in defense responses, a group of identified cDNA clones with differential expression patterns were retrieved from the SSH libraries. Subsequently, their nucleotide sequences were determined. All the resultant sequences were then annotated by comparison of their homology to Genbank databases using the BLAST search program. If necessary, sequences were assembled into contigs to allow assessment of several different sequences (genes) identified. When a strong sequence homology (with a threshold *E* value ≤ 10^−5^) was found to a gene with known function, the function was putatively assigned with confidence to these identified cDNA clones. Then, the genes and their functions were categorized using the molecular functions listed by the Gene Ontology Consortium (GOC). Gene annotations for 111 differentially expressed cDNA fragments are given in Table 2. They were then categorized into 10 major groups (Figure 6), including about 13% of the genes that could not be annotated and therefore were categorized as “unknown”. The two most numerous categories containing annotated gene products were defense-related functions and signal transduction pathways (Figure 6).

### Activation of common defense-related genes and defense-signaling proteins

**FIGURE 7:**
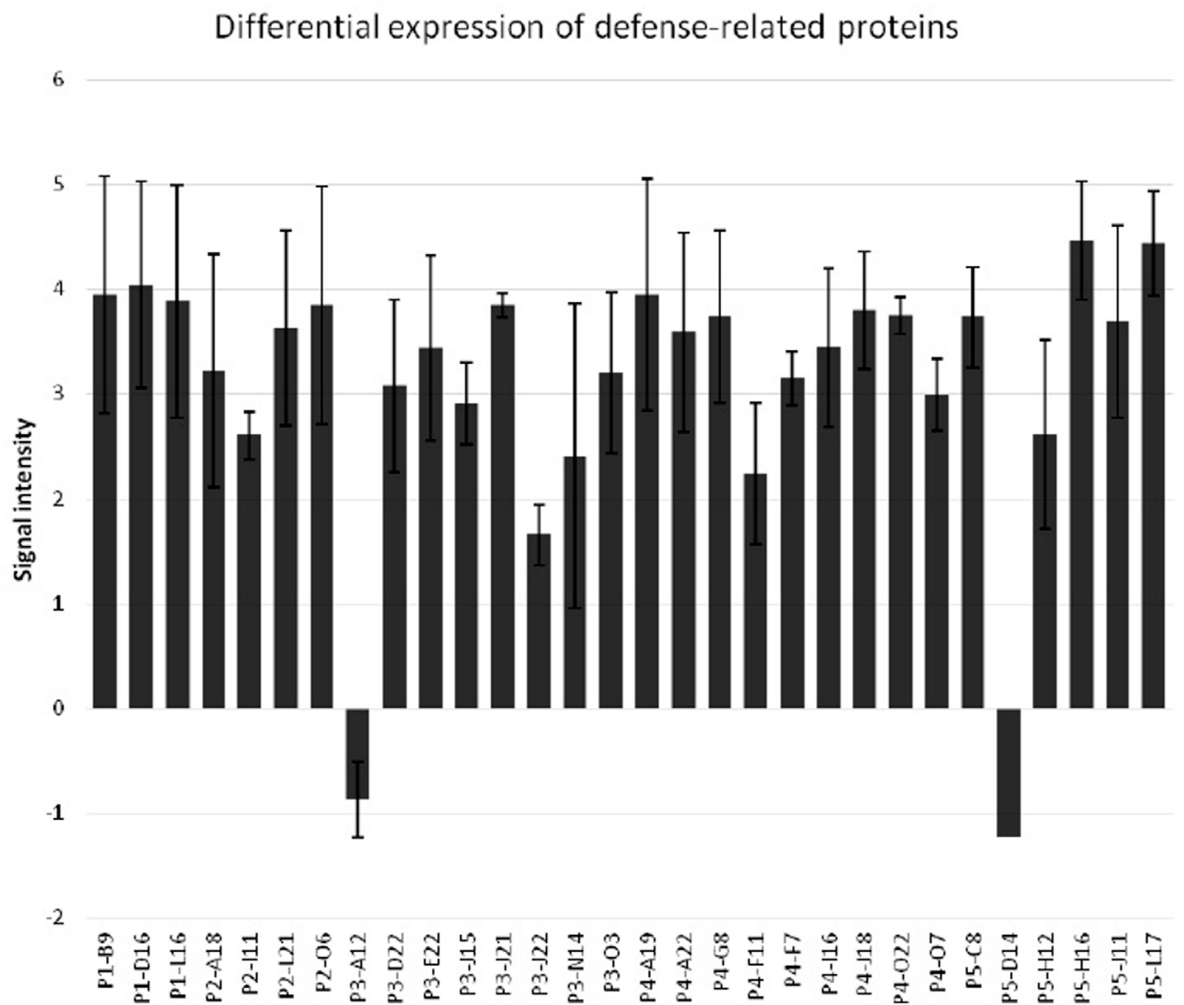
Differential expression of defense-related proteins in sorghum seedlings upon the infestation by greenbug. The *y*-axis represents the ratios of signal intensity of greenbug-induced or suppressed genes. The *x*-axis shows various defense-related genes of sorghum.

**FIGURE 8:**
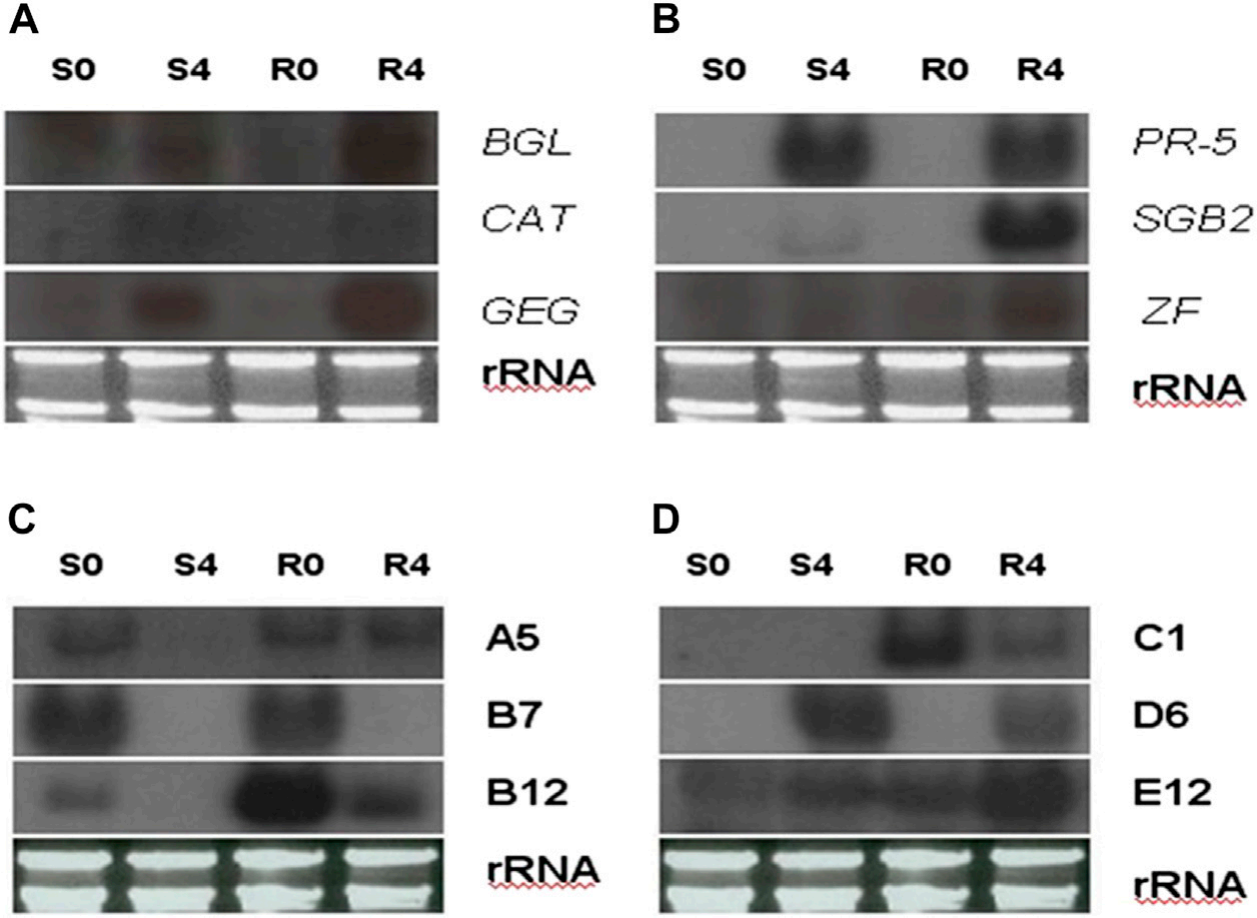
Northern blot analysis shows transcript abundance of four representative greenbug-regulated cDNA clones identified by cDNA microarray. In the blots, RNA samples were arranged from left of each blot as lane S0, un-infested susceptible plants; S4, susceptible plants infested by greenbug for 4 days; R0, un-infested resistant plants; and R4, resistant plants infested by greenbug for 4 days. In **(A,B)**, the specific probes used in the Northern blots were the genes encoding for β-1,3-glucanase (*BGL*), catalase (*CAT*), glucan endo-1,3-glucosidase (*GEG*), pathogenesis-related protein (*PR-5*), sulfur-rich thionin-like protein (*SGB2*), and zinc finger-like protein (*ZF*). In **(C,D)**, the specific probes used in the Northern blots **(C,D)** were these greenbug-responsive transcripts (A5, B7, B12, C1, D7, E12) with unknown function.

While evaluating the transcriptomics profiles, it was noticed that a group of plant defense related genes were activated in response to greenbug attack as shown in Table 3. Those defense genes showed Cy5/Cy3 ratios reproducibly above the 2-fold thresholds in the microarray experiments (Figure 7; Table 2), suggesting their activities were induced by challenge with greenbug. Upregulation of a group of plant defensive genes in the resistant plants (PI 550607) was confirmed by the DEG data from both microarray and RNA-seq, indicating that they have role in plant defense against aphids and also shows the concordance of gene identification between the RNA-seq and microarray platforms (Table 4). Of those upregulated genes, expression of β-1,3-glucanase (BGL) and class III chitinase increased by 3.22 folds and 3.44 folds, respectively, in sorghum seedling tissues at 96 hpi when compared to controls. These increases in expression are consistent with an earlier report, in which the increases of both chitinase and β-1,3-glucanase activities were observed when sorghum plants were exposed to multiple treatments, including insects, fungi and wounding (Krishnaveni et al., 1999). Changes in expression of β-1,3-glucanase revealed by the microarrays were validated by Northern blot analysis (Figure 8). According to the Northern data, although β-1,3-glucanase mRNA levels were induced in both resistant and susceptible genotypes following greenbug infestation, its expression level in the resistant plants was much higher than in susceptible plants. A cDNA coding for a pathogenesis-related protein (PR-5) was also identified, which showed as an upregulated gene (2.9 folds) in greenbug-infested seedlings. Among the greenbug-responsive transcripts, a cDNA encoding a thaumatin-like protein was induced in sorghum seedlings following greenbug attack. Its signal intensity on the microarray was 3-fold higher in greenbug-challenged tissues compared with control plants (Table 2), suggesting its function in defense response against greenbug infestation.

**TABLE 3:** List of plant defensive genes and their roles in host resistance to pest insects. Their transcripts were identified in resistant aphid-resistant genotype (PI 550607) challenged by greenbug. Among the 23 greenbug-induced genes listed below, 12 genes were identified in this resistant genotype of sorghum but not in other studies.

Gene product	Function	Remark^a^
B-1,3-glucanase (BGL) (*PR*-2)	Hydrolyzes callose and glucan polymers	2
Bromelian-like thiol protease	Inhibits the growth of a wide range of insects	unique
Catalase (CAT) (*PR*-3)	H_2_O_2_-scavenging enzyme	1, 2
Chitinase (class III)	Damages the insect midgut	2
Cyanogenic β-glucosidase dhurrinase-2	Produces the hydrogen cyanide toxic to insects	unique
DnaJ-like protein	Cellular protection	unique
GDSL-lipase	Lipid metabolism and signaling	unique
Glucan endo-1,3-B-glucanase	Hydrolyzes glucan polymers	1
Glutathione S-transferase (GST)	Detoxify or inactivate toxic compounds and ROS	1, 2
LHY Protein	Cell maintenance	2
Lipases (family II lipase EXL4)	Lipid metabolism and signaling	unique
Lipoxygenase (LOX)	JA biosynthesis pathway enzyme	2
Mannose Binding Lectin Precursor	Antinutritive effort, carbohydrate-binding proteins	unique
Map kinase phosphatase (MK-STYX)	Signaling, phosphorilation of transcription factors	unique
Methyltransferase	Secondary metabolism - phenylpropanoids	2
Pathogenesis-related protein-5 (*PR*-5)	Defense against diseases	unique
Phosphoethanolamine N-methyltransferase	Osmotic stress defense	unique
Phospholipase A_2_ (PLA_2_)	Signaling, generates second messenger	unique
Polyphenol oxidase PPO1	Deterrents to insects as it reduces nutritive value	unique
Proline-rich protein	Cell wall fortification	1
Sulfur-rich thionin-like protein	Defense against diseases	1
Thaumatin-like protein (α–amylase)	Digestive enzyme inhibitor	2
Zinc finger-like protein	Transcriptional regulator	unique

^a^ Most of these transcripts listed above are unique to the sorghum genotype used in this study; while some transcripts were similar to the results of previous studies:1, Park et al., 2006; 2, Zhu-Salzman et al., 2004.

**TABLE 4:** List of the important genes identified in the resistant genotype (PI 550607), which were upregulated in the plants in response to aphid infestation. Their strong activities were confirmed by multiple sets of data from microarray, RNA-seq, Northern analysis and qPCR tests.

Gene name	Fold change identified by microarray	Fold change identified by RNA-seq	Accession No./Gene id
β-1,3-glucanase (BGL, PR-2)	3.219	2.570	DV162830/SORBI_3002G327900
Catalase (CAT, PR-3)	3.624	-	DV162816
Glucan endo-1,3-β-glucosidase	2.405	-	DV162843
Pathogenesis-related protein-5 (PR-5)	2.906	2.501	DV162815/SORBI_3008G182900
Patatin-like proteins	4.225	2.438	DV162754/SORBI_3005G219000
Sulfur-rich thionin-like protein	3.947	4.032	DV162839/SORBI_3006G007300
Zinc finger-like protein	2.005	3.745	DV162787/SORBI_3006G005400

Note: β-1,3-glucanase (BGL, PR-2) was abbreviated as BGL in the Northern blot, Catalase (CAT, PR-3) as CAT, Glucan endo-1,3-β-glucosidase as GEG, Pathogenesis-related protein-5 (PR-5) as PR5, Patatin-like proteins as SGB1, Sulfur-rich thionin-like protein as SGB2, and Zinc finger-like protein as ZF.

Transcripts for proteins that detoxify compounds present in the greenbug-infested tissues were identified in this expression profile. For example, the level of the transcript for glutathione S-transferase (GST) was induced (2.79 folds) in sorghum seedlings during infestation with greenbugs. This reactive oxygen species (ROS) scavenging enzyme could act as glutathione oxidase, which directly detoxifies radicals (Marrs, 1996; Pant and Huang, 2020). Catalase (CAT), another H_2_O_2_-scavenging enzyme, also showed up in the profiles of sorghum seedlings challenged by greenbugs. The level of catalase transcript was 3.13-fold higher based on the Cy5/Cy3 signal ratio, indicating that induced expression of this enzyme could be a part of the general defense response of sorghum plants against stresses, including greenbug feeding. Indeed, the Northern blot data showed that catalase expression was induced in both genotypes following greenbug infestation (Figure 8).

In addition, several cDNAs annotated in the Genbank database were signaling molecules and regulatory proteins, which included lipoxygenases (LOX), a zinc finger-like protein, a translation initiation factor, and a map kinase phosphatase-like MK-STYX. These genes were expressed differentially between greenbug-challenged and control plants. For example, three clones of zinc finger-like protein showed induction in a range of 2.00 to 3.01 folds by microarray, and the upregulation of the transcript in the resistant genotype was confirmed by Northern analysis (Figure 8). The identification of this set of sorghum genes that were differentially regulated by phloem-feeding aphid suggests their roles in plant defense against greenbug attack. According to sequence analysis, these LOX cDNA clones share similarity to their homologs in different species including maize, rice, barley, and sorghum (Shrestha et al., 2021).

### Specific defense responses to greenbug phloem-feeding in sorghum plants

Greenbug infestation induced some specific host responsive genes. These genes include the SGB1 gene coding for patatin-like proteins, SGB2 for sulfur-rich thionin-like proteins, and cyanogenic β-glucosidase (also named as dhurrinase) based on the annotation. From the analysis of sequenced cDNA clones, the family of Patatin-like protein produced the most abundant transcripts, and sulfur-rich thionin-like protein transcripts were the second. cDNA clones were randomly picked for sequencing before normalization, patatin-like protein had 16 hits and sulfur-rich thionin-like protein had 14 hits (Table 2). It is not surprising that they were the most abundant transcripts because the resistant plants could make a large amount of these defense compounds while fighting against greenbugs.

**FIGURE 9:**
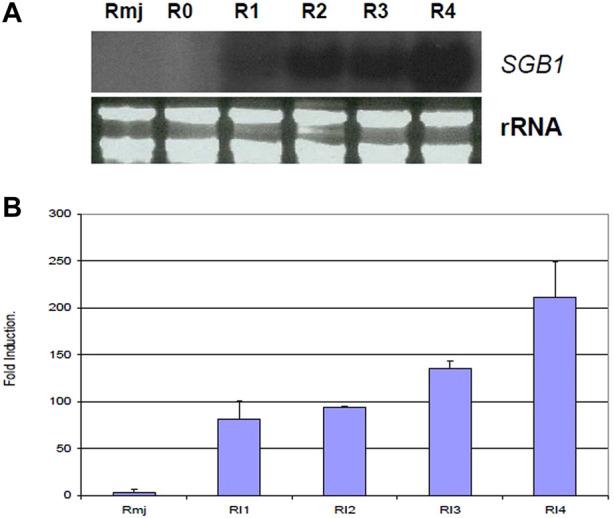
Transcriptional changes of the sorghum *SGB1* gene were analyzed using Northern blotting and qRT-PCR. In the **(A)**, Northern blot depicts expression levels of the *SGB1* gene in resistant plants without infestation (R0), 1 day (R1), 2 days (R2), 3 days (R3) and 4 days (R4) after infestation, and the resistant plants treated with MeJA solution for 24 h (Rmj). In the **(B)**, the graph shows fold changes (mean ± SE) of the *SGB1* transcripts in the resistant sorghum seedlings at 24-hour post-infestation (hpi) with greenbug (RI1), 48 hpi (RI2), 72 hpi (RI3), 96 hpi (RI4), and sorghum seedlings grown at the same conditions which were treated with MeJA for 24 h (Rmj) but no greenbug infestation.

According to signal ratios in microarrays, greenbug induced a substantially high level of the SGB1 expression in the resistant plant. The level of expression ranged from 2.93 to 4.55 folds increase in this gene group (Table 2). Changes in gene expression revealed by the microarrays were validated for selected genes using Northern blot analysis. The SGB1 gene exhibited very strong expression at 72 and 96 hpi in the current study, evidenced by Northern analyses (Figure 9), which is consistent with the microarray data, but SGB1 showed no expression without challenge by greenbugs. SGB1 showed a negligible expression in the susceptible sorghum genotype at the same time point. To determine the regulation of SGB1 expression by other factors such as MeJA, a global signaling molecule, an experiment was carried out to analyze the effect of MeJA on SGB1 expression. Interestingly, the SGB1 gene did not respond to treatment with MeJA. Sorghum seedlings at the same age were treated with MeJA solution (100 µM) for 24 h and RNA was extracted from the seedling tissues for Northern blotting. But the transcripts of SGB1 gene could not be detected in the MeJA-treated tissues, which indicate that expression of the SGB1 gene is MeJA-independent. The induction of SGB1 gene by greenbug was further confirmed using quantitative real-time PCR (qRT-PCR). The gene-specific primers used for the qRT-PCR were CCC​ACC​CAA​TCA​TCG​ACT​TC (forward primer) and GCC​ACC​TTC​TGC​TCA​AGA​AC (reverse primer). The average threshold cycles (*C*_T_) values calculated from triplicate reactions of each time point were used to determine the expression fold changes relative to the control (i.e., 0 time point representing the un-infested tissues). The fold change values are presented for the levels of *SGB1* expression. The sorghum α-tubulin gene was used as an internal control. The qRT-PCR analyses at multiple time points indicated that the transcripts of the SGB1 gene were greenbug-specific and rapidly increased over the time course (Figure 9). The level of SGB1 mRNA had an 81.33-fold increase at 24 hpi and increased continuously to 211-fold higher at 96 hpi in infested plants when compared with the control.

The SGB2 gene encoding sulfur-rich thionin-like protein was induced by greenbug and its product was highly abundant in the greenbug-infested resistant seedling tissues. The accumulation of its transcripts was observed as a 4-fold increase in response to greenbug challenge (Table 2). A probe encoding SGB2 in sorghum was hybridized to the RNAs from various treatments to confirm the microarray results. As expected, the Northern blot result for the SGB2 gene was consistent with the microarray data. Expression of the SGB2 gene was not detected in controls (i.e., non-infested seedlings) of both resistant and susceptible genotypes (Figure 8). However, a strong expression was induced by greenbug in the resistant genotype but was negligible in susceptible plants (Figure 8.3).

A cDNA coding for cyanogenic β-glucosidase was observed in the transcriptional profile of sorghum, and this enzyme catalyzes the generation of hydrogen cyanide (HCN) from a cyanogenic glucoside precursor. The toxic HCN has been generally associated with host plant defense against herbivores and pathogens. According to our microarray data, its transcript accumulated in greenbug resistant sorghum plants following the challenge with greenbug and was 2.24-fold higher in the infested plants compared with control plants at 96 hpi. As shown in Table 2, sorghum β-glucosidase gene (glucan endo-1,3-β-glucosidase, GEG) also responded to greenbug attack, whose transcripts were relatively abundant (2.41 folds) in resistant sorghum seedling following greenbug infestation. Induction of GEG in the resistant genotype was much stronger than in susceptible plant as demonstrated by Northern analysis (Figure 8). We also noticed that aphid feeding caused a strong induction (3.2-fold) of polyphenol oxidase (PPO) expression in resistant sorghum plants, suggesting its defensive role against greenbug aphid. Another plant defense compound that was also detected in the differential expression profile was mannose binding lectin precursor (Table 2). Upregulation of this anti-nutritive protein is an important factor in combating harmful aphids.

### Unknown defense factors

It is not surprising that deduced amino acid sequences from some of the greenbug-responsive transcripts show no similarities with any of known proteins. A few of the cDNAs from this group were analyzed using Northern blotting. Their differential expression patterns were apparently regulated by greenbug (Figures 8C, D), suggesting that these expression products were involved in the interactions between host plants and greenbug. However, confirmation of the role of those unknown genes during host response to greenbug challenge requires further functional characterization. Information obtained from further characterization may lead to the identification of novel genes important for host defense and will probably contribute to a better understanding of other aspects of host response or resistance.

## Discussion

In the natural ecosystem, herbivorous insects and their higher plant hosts make up a massive portion of the earth’s biodiversity and biomass. Plant-insect interactions are always dynamic. As a result, many plants have evolved highly sophisticated defense machinery to protect themselves from attack by enemies such as insect pests. Genetic variations for resistance to herbivores and microbial pathogens are widespread in plant populations where the host plants deploy a wide variety of strategies in combating pests (Fidantsef et al., 1999; Walling, 2000). Interactions between plants and insects are complicated, resulting in marked changes in gene expression that contribute to host defense and tissue repair (Reymond et al., 2000). To characterize molecular responses to cereal aphids in greenbug-resistant sorghum plants, we performed genomic-wide gene expression analysis in sorghum seedlings challenged by greenbug. Our results from this study are consistent with the general strategy of host defense against pathogens and herbivores (Fidantsef et al., 1999; Walling, 2000). In response to greenbug phloem-feeding, sorghum plants initiated a large transcriptional reorganization (Park et al., 2006; Grover et al., 2022). The gene expression profile of sorghum response to greenbugs allowed identification of 459 differentially expressed gene products, representing 26.1% of 1761 cDNA sequences printed on the custom-designed arrays. The data also confirmed the idea that complex defense strategies were deployed in sorghum seedlings to protect themselves against greenbug attack. The defensive strategies include the rapid transcriptional activation of defense genes (Figure 7; Table 3) and the production of a multitude of defensive chemicals against their enemies. On the other hand, it is interesting to note that relatively few genes were found to be downregulated in the greenbug-infested resistant seedlings (Figures 2, 7). The resistant sorghum genotype has an effective defense system against greenbug and fully protected itself. Thus, most physiological activities had not been affected negatively.

### Roles of common defense mechanisms and signaling network in host defense

Functional annotation extracted from public databases revealed that many of the sorghum transcriptional responses were associated with cell maintenance and cell wall fortification, metabolism and development, oxidative stress, defense related products, and signal transduction molecules (Table 2). Stress response proteins included lipoxygenases (LOX), glutathione S-transferases (GST), catalases, and DnaJ-like protein. Some of these enzymes were induced in the greenbug-challenged seedling tissues probably because during feeding, aphid stylets and salivary secretions altered oxidative/osmotic conditions, or plant oxidative stress was triggered by altered phloem flux and disturbance of cell walls (Pollard, 1973). It is not surprising that many stress-responsive proteins can be induced by biotic and abiotic influences, including herbivores, and may be essential for establishment of cellular stress tolerance. The fact that genes showed similar expression profiles in response to wounding does not imply that they are regulated by the same signals (Reymond et al., 2000).

Genes encoding for cell maintenance and cell wall fortification enzymes were also differentially expressed in response to greenbug infestation. For example, transcripts of genes coding for proline-rich protein (PRP) were highly abundant in greenbug-feeding seedling tissue. DNA sequence analysis indicates that sorghum PRP consists of a highly repetitive proline-rich sequence and is presumably involved in the formation of secondary cell walls in general. PRP is one of the basic structural components of plant cell walls and appears to be integrated with the remodeling of the plant cell wall during the defense response. Insolubilization of PRP is generally intensified in response to wounding and treatment with pathogenic elicitors (Fowler et al., 1999). This insolubilization is considered to be involved in acceleration of further fortification of cell walls (Showalter, 1993). In response to insect feeding, cell wall reinforcement was induced by accumulation of PRP, lignin, hydroxylproline-rich glycoprotein (HRGP) and extensin in addition to synthesis of other defense molecules (Kim et al., 2002). Since greenbugs feed by inserting their stylet-like mouthpart into the plant tissue and sucking out plant juices, cell wall fortification may occur rapidly to protect plant cells against greenbug feeding in addition to other early responses, including ion fluxes, production of reactive oxygen intermediates (ROIs), and subsequent synthesis of defense molecules (Bouché et al., 2005). Accordingly, several genes associated with oxidative stress, including catalases and glutathione S-transferases (GST), were upregulated during the interaction between resistant sorghum plants and greenbugs. The activation of specific cellular protection mechanisms is likely to accompany the defense response, which minimizes the consequences of oxidative stress.

Several signal transduction genes were upregulated by greenbug attack. Phospholipase A_2_, GDSL-lipase, lipoxygenases (LOX), and Map kinase phosphotase all accumulated in the leaves of resistant sorghum seedlings in response to greenbug feeding. Rapid increases in the level of LOX mRNA and lipoxygenase (LOX) enzyme activities are frequently found to be specifically associated with feeding of herbivores (Jonak et al., 2002; Shrestha et al., 2021). Increased activity of GDSL-lipase may also contribute to resistance to greenbug. Both GDSL-lipase and LOX generate signal molecules such as JA, methyl-JA, or lipid peroxides, which coordinately regulate expression of defense genes. Among the regulation factors, transcripts of MAP kinase phosphatase (often called MKP) were also detected, indicating its role in several cellular signaling processes in response to environmental signals, such as biotic and abiotic stresses. Our microarray analyses have also captured transcripts associated with regulation of gene expression. These regulators included transcriptional factors such as zinc finger-like proteins and homeobox protein Hox, LHY protein, and a homolog of translation factor 2 alpha subunit eIF2 of Arabidopsis, suggesting that these factors in sorghum play certain roles in host cells during plant-aphid interactions. We have detected a putative LHY protein gene from the greenbug-responsive expression profile. The gene encodes a Myb-related DNA binding protein which is believed to be generally regulated by dehydration, insect feeding, and wounding (Lan et al., 2005).

Challenge by greenbug on resistant sorghum seedlings stimulated expression of pathogenesis-related (PR) proteins and other defense-related proteins, including PR-5, chitinase, and β-1,3-glucanase. According to Stout et al. (1994), insect herbivores often induce PR proteins in their host plants because of feeding. Transcripts of PR proteins were documented in the induced expression profiles of *Arabidopsis thaliana* plants infested by aphids (Kessler and Baldwin, 2002), and of tomato plants infested by whiteflies (Mayer et al., 1996; Walling, 2000). In addition, both β-1,3-glucanase and chitinase were induced in a susceptible sorghum genotype (Zogli et al., 2020) but their expressions were stronger in this resistant sorghum genotype (PI 550607). PR proteins possess either antifungal or antimicrobial activities and can degrade the cell walls of microbial cells. Although their functions are well known in disease resistance, little is known about the effect of PR proteins on host defense against herbivores. It has been reported that PR proteins were induced as a general defense response in the host plants, thus their activities could be detected following various stresses, including phloem-feeding insect, whitefly (Soliman et al., 2019), suggesting that PR proteins do play roles in plant’s defense against insects, including phloem-feeding aphids.

### Identification of genes directly involved in host defense against greenbug aphids

Although plant defense responses to various stresses such as microbial infection, insect challenge feeding, and wounding are overlapping (Inbar et al., 1998), studies have revealed certain responses that were induced specifically or activated more rapidly by insect feeding (Reymond et al., 2000). In this study, differences have been observed in the expression patterns of several aphid-induced genes. For example, the SGB1 gene is clearly one of the greenbug responsive genes, which was activated in sorghum plants by greenbug feeding. The amino acid comparison of the SGB1 sequence suggested that it encodes a patatin-like protein (PLP). The sorghum patatin-like protein shares conserved catalytic domain of plant phospholipase A_2_ (PLA_2_) proteins and contains an active site (Ser-His-Asp triad) of lipolytic enzymes (Schrag and Cygler, 1997). Plant phospholipase A_2_, associated with lipolytic activity, has been shown to function in plant signal transduction, which was evidenced by the report that PLA was rapidly activated by a plant-pathogen interaction (Holk et al., 2002). The rapid accumulation of sorghum PLA_2_ transcripts in greenbug-challenged resistant sorghum seedling tissues is consistent with those previously reported results (Holk et al., 2002). When measured with a sensitive real-time PCR assay as shown in Figure 9, the level of PLA_2_ expression was found to be 83-fold higher at 24-hour post infestation than in non-infested tissues and reached a 211-fold increase at 96 hpi. In another example, the similar trend of PLA_2_ activity was also observed in tobacco mosaic virus (TMV)-infected tobacco plants (Dhondt et al., 2000). In TMV-infested tobacco leaves, PLA_2_ activity rose dramatically between 36 and 72 h following virus infection, and near-maximal levels were reached by 96 h. Our results show that expression of sorghum PLA_2_ was not induced by jasmonic acid treatment (Figure 9). In general, genes involved in the synthesis or metabolism of members of the jasmonate family (e.g., LOX and AOS) are coordinately induced by wounding and MeJA treatment. Considering the data from our studies and other reports (Walling, 2000), it is believed that the sorghum PLA_2_ gene is specifically induced in sorghum by greenbug feeding, but not by wounding. Moreover, molecular characterization of the sorghum PLA_2_ gene and regulation of its expression suggest that this gene, like other plant PLA_2_ genes, may play an important role in plant signal transduction and contribute to host defense against greenbug attack in sorghum plants.

Another example for greenbug-responsive genes identified in this study is SGB2, which encodes a sulfur-rich thionin-like protein. The sorghum SGB2 gene is strikingly similar in amino acid sequences to the purothionins from wheat endosperm (Colilla et al., 1990). Its expression pattern is like the sorghum SGB1 (PLA_2_) gene. The SGB2 transcript was strongly induced and highly abundant in greenbug-challenged resistant sorghum plants, but not detectable in unchallenged plants, suggesting that the SGB2 gene is specifically activated in response to greenbug attack. This observation was revealed in the microarray (Table 2) and confirmed by Northern blot analysis (Figure 8). Activity of the SGB2 gene specifically in greenbug-challenged sorghum tissues provided the first indication of its function in the host defense against the greenbug pest. Previous studies have already demonstrated that sorghum thionins (sorghum inhibitors) possess inhibitory activity to insect α-amylases (Bloch and Richardson, 1991). The members of these types of insect α-amylase inhibitors have also been isolated from other cereal crops, including wheat and barley. The groups of sulfur-rich (or cysteine-rich) thionin proteins, which are known to be toxic to insect larvae (Jones et al., 1985), are thought to form a part of the plant protective mechanisms against insect and pathogens.

Among genes likely to be directly involved in defense against insects, we observed the upregulation of a cDNA coding for cyanogenic β-glucosidase dhurrinase-2 (*Dhr*) in greenbug-challenged resistant sorghum seedlings. We believe this cDNA clone is the *Dhr* gene as its sequence showed a high similarity to the sorghum *Dhr* gene previously isolated from sorghum seedlings (Cicek and Esen, 1998). Cyanogenic β-glucosides (dhurrin), a plant secondary metabolite, have long been known to play an important role in host defense against herbivore attack (Hruska, 1988). In fact, cyanogenic β-glucosides are widely distributed in many plant species and present in relative high level in sorghum plants. Hydrogen cyanide is generally stored in plant tissues in a nontoxic form, often combined with a sugar to form a cyanogenic β-glucoside. When plant tissue is disrupted by herbivore attack, the cyanogenic glucosides degrade into sugar, releasing the respiratory poison hydrogen cyanide (HCN) upon hydrolysis by dhurrinase. Thus, the production of cyanogenic glucosides appears to be a natural defense mechanism in sorghum plants to protect themselves from insect attack. It is of interest to note that the pathway for biosynthesis of the cyanogenic β-glucoside dhurrin has been successfully transferred from sorghum into an acyanogenic model plant *A. thaliana* using genetic engineering. That experiment has demonstrated that the transgenic *A. thaliana* expressing dhurrin acquired resistance to the flea beetle (Tattersall et al., 2001). Our results imply that expression of dhurrin in sorghum plants could be a part of the resistance response to greenbug, although direct evidence would be helpful to confirm this fact.

From the profile of greenbug-induced genes, we also noticed that a transcript representing mannose binding lectin precursor was induced to a moderate level. The induction of lectin during the sorghum-greenbug interaction suggested its role in host defense. Lectins are well characterized sugar-binding proteins, many of which have insecticidal properties (Peumans and Van Damme, 1995) thought to be based on specific binding to glycoproteins in the insect gut. The activity of lectins against insect has been reported in several previous studies. Gatehouse et al. (1999) demonstrated insecticidal activities of a glucose/mannose-binding lectin (i.e., concanavalin A) from Jacobean (*Canavalia ensiforms*) on two crop insect pests, tomato moth (*Lacanobia oleracea*) and peach-potato aphid (*Myzus persicae*). When feeding on transgenic potato plants expressing concanavalin A, the fecundity of peach-potato aphids decreased by 45%. Greenbug is closely related to the peach-potato aphid, and both belong to the Aphididae. Therefore, it is reasonable to assume that lectin gene expression and the activation of lectin production in greenbug-challenged sorghum tissues is one of the protective strategies operating in greenbug-resistant sorghum plants.

Induction of polyphenol oxidase (PPO) mRNA in response to greenbug feeding was also detected in sorghum seedlings. PPO is an enzyme that catalyzes the oxidation of phenolic compounds to ο-quinones. Thus, the PPO enzyme is responsible for the typical browning of plant extracts and damaged tissues caused by the spontaneous polymerization and crosslinking of the *ο*-quinones (Constabel et al., 2000). Felton et al. (1989) has demonstrated that activation of foliar oxidases by insect feeding reduced nutritive quality of dietary proteins of foliage for noctuid herbivores. When PPOs are expressed in mesophyll tissues, they can covalently modify and cross-link dietary proteins during insect feeding, thereby decreasing the digestibility of plant proteins in the herbivore gut (Baldwin and Preston, 1999). Therefore, the foliar PPO of sorghum may also play a role in defense against the phloem-feeding aphid, greenbug.

### Sorghum plants adopt diverse resistant mechanisms and complicated defensive responses to greenbug

Sorghum plants exhibit varied responses to greenbug infestation (Harvey et al., 1991; Burd, 2002), and they likely have different mechanisms or genes for resistance to insect pests although various plants may use a similar strategy to defend different attackers (Mello and Silva-Filho, 2002). The levels of resistance to greenbugs in cultivated sorghum varieties are quite low and only a few greenbug-resistant sorghum genotypes are available. Thus, research efforts have been made to identify sources of resistance in wild relatives of sorghum (Harvey et al., 1991; Kofoid et al., 1991; Andrews et al., 1993; Grover et al., 2019). Recently, we have identified some new sources of resistance after systematic evaluation of over 30,000 sorghum germplasm accessions from various geographic locations (Huang, 2011). We selected a set of 26 greenbug resistant accessions (genotypes) from twelve countries and investigated their genetic diversity with AFLP markers. Our results indicate that relatively diverse forms of greenbug resistance exist in the sorghum collection (Wu et al., 2006). These data also suggest that the resistance from germplasm originating from different geographic regions around the world may be controlled by different loci or different alleles within the same locus.

The recent development of high throughput gene expression profiling technology has made it possible to monitor genome-wide changes in gene expression in response to any biological stress. In crop-aphid interaction, sorghum has received considerable attention lately and may serve as an excellent system for gene discovery associated with crop plant defense against aphid pests. A sorghum cultivar derived from a cross between ATx399 and RTx430, susceptible to greenbug like many other commercial cultivars, was examined for its transcriptional responses to greenbug infestation (Zhu-Salzman et al., 2004). They reported 23 greenbug-regulated transcripts from a microarray consisting of 672 cDNAs based on an expression fold-change ratio cutoff of 1.5. Using a similar approach, our recent study (Park et al., 2006) was conducted with a commercial cultivar (M627 from Mycogen) which is highly resistant to greenbug biotype I. Based on a higher cutoff ratio (≥1.8), a total of 157 transcripts were identified from the cDNA microarray containing 3508 cDNA clones. This study aimed at examining the molecular responses of a wild germplasm line of sorghum to challenge by greenbug. It was observed that 459 transcripts showed ≥2.0-fold changes of expression (including induced or suppressed), representing 26.12% of 1761 SSH inserts printed on the cDNA microarray. Comparison of the responses to greenbug feeding among three different hosts, including the susceptible commercial cultivar, a resistant commercial cultivar, and the wild relative of sorghum, showed that all hosts were able to activate general responsive mechanisms, such as transcripts responsible for oxidative stress, cell maintenance and cell wall fortification, and signal transduction networks. Greenbug aphids elicited a few genes common in all three treatments (Pollard, 1973) and there were more overlapping of responsive genes (Fidantsef et al., 1999) between two resistant genotypes (Figure 5). It is noticeable that extensive differences exist in greenbug-responsive transcripts between resistant and susceptible hosts (Zhu-Salzman et al., 2004; Park et al., 2006). Moreover, when comparing the transcriptional responses of cultivated sorghum genotypes to the wild sorghum genotype, certain novel responses were observed exclusively in the wild sorghum genotype (Table 3). The differential responses of these hosts imply that they have different defense mechanisms or protection strategies against greenbug attack.

## Conclusion

Our results clearly show that molecular responses of host plants to phloem-feeding greenbugs are comprehensive and dramatic changes (up- and downregulation) of gene expression in infested tissues in response to greenbug resulted in immediate activation of the signal transduction pathways and subsequent production of resistance factors for their defensive actions. Furthermore, comparison of the greenbug-induced transcript profiles in this wild genotype with those of greenbug-infested commercial sorghum cultivars provided new insight into the diverse defense mechanisms protecting crop plants from attack by these damaging cereal aphids. The picture of molecular responses to phloem-feeding aphids that emerges from both of the early report (Park et al., 2006) and this study and the other reports mentioned above, is complex. However, data describing molecular interactions between host plants and aphids have begun to accumulate. The development of a complete picture includes identification of both known and putative defense responsive genes and the regulatory network of host defense. Continued efforts to examine host responses taking place in different tissues and/or at various time points will provide more details about those defensive components and molecular events leading to protection from aphid attack. Further development of comprehensive databases of plant-insect interactions and more efficient methods for determining physiological function of unknown genes identified from transcript profiles will enhance the application of this approach in expanding our understanding of interactions between plants and phloem-feeding aphids, as well as regulation of host plant defense against aphid pests.

## Data Availability

The data presented in the study are deposited in the NCBI Sequence Read Archive (https://www.ncbi.nlm.nih.gov/sra), accession number: PRJNA1003060.
