# Supplementary material for: Analysis of plant expression profiles revealed that aphid attack triggered dynamic defense responses in sorghum plant
Source: Front Genet. 2023 Aug 15;14:1194273. doi: 10.3389/fgene.2023.1194273 (PMC10465342; doi:10.3389/fgene.2023.1194273)
Supplement: Supplementary file 1 [file DataSheet1.PDF]

Supplementary Information for the manuscript:

Analysis of transcriptome profiles revealed that aphid attack triggered dynamic defense responses in sorghum plant

Yinghua Huang[1✉], Jian Huang[2¶]

✉ Correspondence: Yinghua.Huang@usda.gov

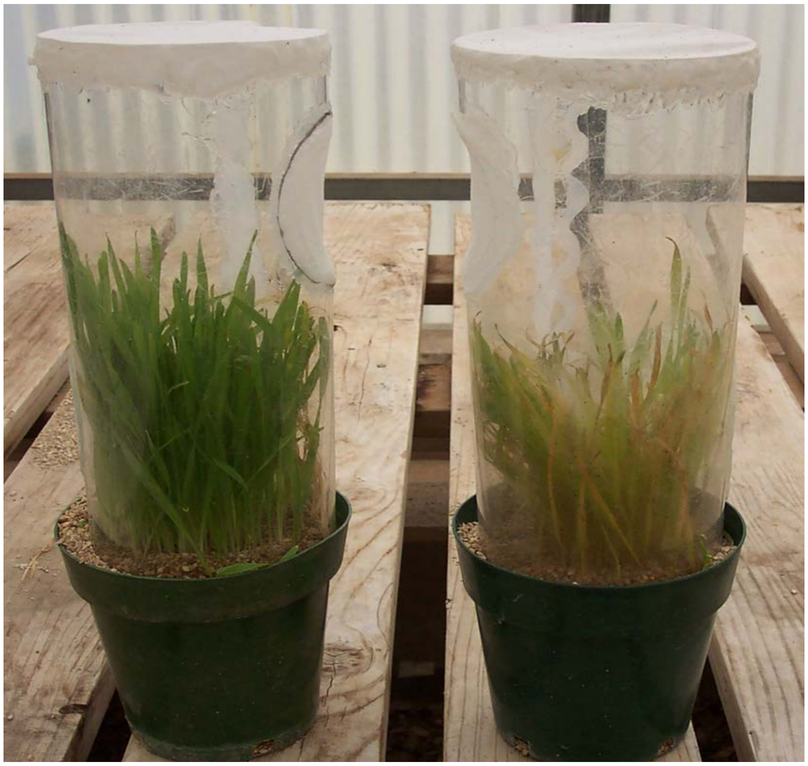

Supplementary Figure S1

Phenotypes of two sorghum genotypes, PI 550607 (resistant, left) and Tx 7000 (susceptible, right) were photographed after co-cultivation with greenbug aphids for 6 days.

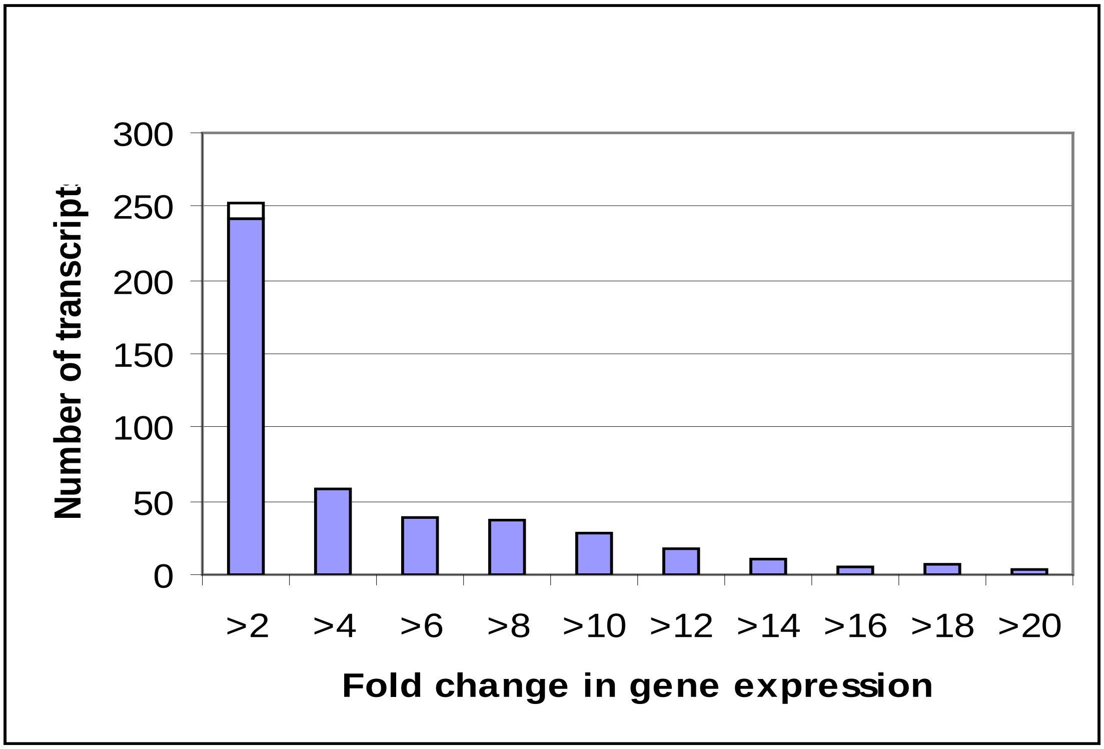

Supplementary Figure S2

Numbers of greenbug-regulated transcripts at various expression levels. The x-axis represents the number of genes and the y-axis represents the range of fold changes of expression compared to control. Shade bars correspond to up-regulated genes and white bars correspond to down-regulated genes.
